# *CaLecRK-S.5*, a pepper L-type lectin receptor kinase gene, confers broad-spectrum resistance by activating priming

**DOI:** 10.1093/jxb/erw336

**Published:** 2016-09-19

**Authors:** Joo Yong Woo, Kwang Ju Jeong, Young Jin Kim, Kyung-Hee Paek

**Affiliations:** Department of Life Sciences, Korea University, Seoul 02841, Republic of Korea

**Keywords:** Broad-spectrum resistance, CaLecRK-S.5, L-type lectin receptor kinase, priming, RNA sequencing, SAR, tobamovirus.

## Abstract

In Arabidopsis, several L-type lectin receptor kinases (LecRKs) have been identified as putative immune receptors. However, to date, there have been few analyses of LecRKs in crop plants. Virus-induced gene silencing of *CaLecRK-S.5* verified the role of *CaLecRK-S.5* in broad-spectrum resistance. Compared with control plants, *CaLecRK-S.5*-silenced plants showed reduced hypersensitive response, reactive oxygen species burst, secondary metabolite production, mitogen-activated protein kinase activation, and defense-related gene expression in response to *Tobacco mosaic virus* pathotype P_0_ (TMV-P_0_) infection. Suppression of *CaLecRK-S.5* expression significantly enhanced the susceptibility to *Pepper mild mottle virus* pathotype P_1,2,3_, *Xanthomonas campestris* pv. *vesicatoria*, *Phytophthora capsici*, as well as TMV-P_0_. Additionally, β-aminobutyric acid treatment and a systemic acquired resistance assay revealed that *CaLecRK-S.5* is involved in priming of plant immunity. Pre-treatment with β-aminobutyric acid before viral infection restored the reduced disease resistance phenotypes shown in *CaLecRK-S.5*-silenced plants. Systemic acquired resistance was also abolished in *CaLecRK-S.5*-silenced plants. Finally, RNA sequencing analysis indicated that *CaLecRK-S.5* positively regulates plant immunity at the transcriptional level. Altogether, these results suggest that *CaLecRK-S.5*-mediated broad-spectrum resistance is associated with the regulation of priming.

## Highlight

*CaLecRK-S.5*, a positive regulator of priming, plays a critical role in plant immunity to broad-spectrum pathogens.

## Introduction

Plants are constantly threatened by a broad spectrum of pathogens in nature. To survive in the presence of these pathogens, they have evolved multilayered immune systems that recognize pathogens at various infection stages. The first layer of plant immunity, referred to as pattern-triggered immunity (PTI), relies on the detection of pathogen-associated molecular patterns (PAMPs) by cell surface-localized pattern recognition receptors (PRRs). Successful pathogens have evolved the ability to avoid or subvert PTI by secretion of effector proteins to the apoplast or into the plant cells. Plants have developed intracellular receptors that recognize effectors directly or indirectly and then activate effector-triggered immunity (ETI) (Jones and Dangl, 2006; Boller and Felix, 2009; Dodds and Rathjen, 2010; Sohn *et al.*, 2014). The two layers of immunity share common defense mechanisms such as reactive oxygen species (ROS) burst, activation of mitogen-activated protein kinase (MAPK) cascades, biosynthesis of antimicrobial metabolites, trigger of transcriptional reprogramming, and in some cases, hypersensitive response (HR) in infected cells (Lorrain *et al.*, 2003; Boller and Felix, 2009; Tsuda and Katagiri, 2010; Segonzac *et al.*, 2011).

In addition to PTI and ETI, plants can activate systemic acquired resistance (SAR) and induced systemic resistance (ISR) after pathogen challenges. SAR is induced by necrotizing pathogens, effectors, or PAMPs and requires salicylic acid (SA), pipecolic acid (PA), and azelaic acid (AZA) (Fu and Dong, 2013; Conrath *et al.*, 2015). On the other hand, ISR is activated by root interactions with growth-promoting bacteria or fungi and requires jasmonate (JA) and ethylene (ET) signaling pathways (Van Wees *et al.*, 2008). Both SAR and ISR are preconditioned states mediated by previous infection or treatments and induce broad-spectrum, long-lasting, and strong defense responses against subsequent challenge by pathogens (Durrant and Dong, 2004; Conrath *et al.*, 2006, 2015; Van Wees *et al.*, 2008). The mechanisms underlying these two key plant systemic immunities largely rely on priming (Conrath *et al.*, 2006). Priming in plant defense is a physiological process by which the plant prepares to respond more quickly or aggressively to future biotic or abiotic stress (Conrath *et al.*, 2015). Priming is induced in local tissue exposed to PAMPs, damage-associated molecular patterns (DAMPs), wound stimuli, pathogen effectors, or chemical compounds such as β-aminobutyric acid (BABA) and in untreated systemic tissue of the plant (Conrath *et al.*, 2006, 2015; Beckers and Conrath, 2007; Chassot *et al.*, 2008; Jung *et al.*, 2009). In the primed state, plants respond to biotic stress with more rapid and robust activation of defense, which is often associated with local and systemic immunity (Conrath *et al.*, 2015). Primed plants show broad-spectrum disease resistance, suggesting that the mechanism of priming partially relies on augmented expression of PTI. However, both SAR- and ISR-induced plants show reduced lesion formation against avirulent pathogens, suggesting that the components of ETI are also involved in priming (Ahmad *et al.*, 2010). Although the accumulation of defense signaling components and epigenetic modifications before exposure to stress are suggested to be involved in priming (Fu and Dong, 2013), the molecular mechanisms underlying priming remain largely elusive.

TMV is a positive-sense single‐stranded RNA (ssRNA) virus and the type species of the genus *Tobamovirus*. TMV causes mosaic disease and reduces the yield of tobacco, tomato, cucumber, and pepper (Scholthof, 2004). *Nicotiana* plants that carry the *N* disease resistance gene, which encodes a class of R protein that contains the Toll/interleukin-1 receptor homology domain, a nucleotide binding site, and a leucine-rich repeat site (TIR-NB-LRR), recognize the helicase domain of the TMV replicase protein to induce HR and restriction of viral replication (Marathe *et al.*, 2002; Martin *et al.*, 2003). On the other hand, in pepper plants, *L* gene alleles specify resistance to TMV infection. *L* gene alleles encode coiled-coil type (CC-NB-LRR) resistance proteins and are categorized into four classes: *L*^*1*^, *L*^*2*^, *L*^*3*^, and *L*^*4*^ (Boukema, 1980; Tomita *et al.*, 2008, 2011). *Capsicum annuum* L. cv. Bugang carries the *L*^*2*^ gene, which recognizes the coat protein (CP) of TMV-P_0_ to induce HR and restrict the virus at local infection sites. *Pepper mild mottle virus* pathotype P_1,2,3_ (PMMoV-P_1,2,3_) can avoid recognition by the *L*^*2*^ gene and thereby spread systemically in *C. annuum* L. cv. Bugang (Berzal-Herranz *et al.*, 1995; de la Cruz *et al.*, 1997; Tsuda *et al.*, 1998; Genda *et al.*, 2007; Hamada *et al.*, 2007; Antignus *et al.*, 2008; Huh *et al.*, 2015). The Gram-negative bacterial pathogen *Xanthomonas campestris* pv. *vesicatoria* (*Xcv*) and the oomycete pathogen *Phytophthora capsici* cause bacterial spot disease and blight disease, respectively, on pepper (Davison, 1998; Jones *et al.*, 1998). An avirulent strain of TMV, *Xcv*, and putative PAMPs of *Phytophthora* induce priming or SAR in plants, and the primed plants show effective resistance to secondary infection (Ross, 1961; Siegrist *et al.*, 1998; Kang *et al.*, 2005; Lee and Hwang, 2005).

LecRKs are a group of receptor-like kinases (RLKs) with an extracellular legume-like lectin domain, a transmembrane domain (TM), and an intracellular kinase domain. Arabidopsis has 45 *LecRK*s, which are divided into nine clades and seven singletons (Bouwmeester and Govers, 2009). Several *LecRKs* are involved in response to biotic and abiotic stresses. One of these is *LecRK-I.9*, which maintains cell wall–plasma membrane continuum and plays a crucial role in disease resistance to *Phytophthora* in Arabidopsis, *Nicotiana benthamiana*, and potato (Gouget *et al.*, 2006; Bouwmeester *et al.*, 2011, 2014). Recently, *LecRK-I.9* was identified as a receptor of extracellular ATP (eATP), which is released by wounding stress or pathogen infection (Choi *et al.*, 2014). *LecRK-V.5* represses stomatal immunity against bacterial pathogens but positively regulates disease resistance against *Phytophthora* (Desclos-Theveniau *et al.*, 2012; Wang *et al.*, 2014). *LecRK-VI.2* is suggested to be essential for BABA-mediated priming and positively regulates flg22-induced PTI by interacting with the flagellin receptor FLAGELLIN SENSING2 (FLS2) (Singh *et al.*, 2012; Huang *et al.*, 2014). The overexpression of *LecRK-IV.3* in Arabidopsis shows increased disease resistance to *Botrytis cinerea* and enhanced seed germination under high-salinity conditions (Huang *et al.*, 2013). Systematic functional screening of Arabidopsis *LecRK* T-DNA insertion lines with *Alternaria brassicicola* and *Phytophthora* or *Pseudomonas* pathogens suggested that additional *LecRKs* have functions in immunity against these pathogens (Wang *et al.*, 2014). However, the extent to which *LecRKs* function in disease resistance in crops remains largely unknown (Wang *et al.*, 2015, 2016).

In this study, we identified, by microarray analysis, a *C. annuum* L-type lectin receptor kinase gene, *CaLecRK-S.5*, induced by TMV-P_0_ infection. *CaLecRK-S.5*-silenced pepper plants showed significant reduction of defense responses to TMV-P_0_. In addition, we showed that *CaLecRK-S.5* confers broad-spectrum resistance against a virulent PMMoV-P_1,2,3_ strain and bacterial and oomycete pathogens. A BABA treatment experiment revealed that priming is involved in these *CaLecRK-S.5*-mediated resistance responses. Bacterial pathogen-mediated SAR was also abolished in *CaLecRK-S.5*-silenced plants. Finally, RNA sequencing was performed to investigate transcriptional reprogramming in *CaLecRK-S.5*-silenced plants and showed that *CaLecRK-S.5*-mediated priming plays a positive role in plant immunity.

## Materials and methods

### Biological materials, growth conditions, and pathogen inoculation

Pepper (*C. annuum* L. cv. Bugang and *C. annuum* L. cv. Nockwang) and *N*. *benthamiana* plants were grown under 24–26 °C day and 17–19 °C night temperatures under a 16-h light:8-h dark cycle. For viral infection assays, approximately 5-week-old plants were inoculated with TMV-P_0_ (avirulent) and PMMoV-P_1,2,3_ (virulent) strains as described previously (Ham *et al.*, 2006; Huh *et al.*, 2015). To monitor systemic responses, the fifth or sixth leaf was inoculated with virus-containing sap and upper leaves were harvested at 9 or 14 d post-inoculation (dpi). For *Phytophthora* infection assays, *P*. *capsici* was grown on V8 vegetable juice agar plates at 28 °C. *Phytophthora* spores were harvested as described previously (Kamoun *et al.*, 1998) and diluted to 50 000 spores ml^−1^. Wet cotton containing *P*. *capsici* spores was applied to abaxial sides of 5-week-old plant leaves, and the plants were kept for several days under 100% relative humidity. For *Xanthomonas* inoculation assays, approximately 5-week-old plants were inoculated with the *Xcv* virulent strain Ds1 (*Xcv* Ds1) and the avirulent strain Bv5-4a (*Xcv* Bv5) as described previously (Lee and Hwang, 2005). Bacterial suspensions were adjusted to a concentration of 10^6^ colony-forming units (cfu) ml^−1^ for *Xcv* Ds1 inoculation and 10^7^–10^8^ cfu ml^−1^ for *Xcv* Bv5 inoculation. For the SAR assay, primary leaves pretreated with *Xcv* Bv5 (10^7^ cfu ml^−1^) were incubated for 2 d and their upper leaves were inoculated with *Xcv* Ds1 (10^6^ cfu ml^−1^). Bacterial growth was determined at 0 and 3 dpi.

### RNA isolation, reverse transcription polymerase chain reaction (RT-PCR), and quantitative real-time RT-PCR (qRT-PCR) analysis

Total RNA was isolated from pepper and *N*. *benthamiana* leaf tissues using an RNeasy Mini kit (Qiagen, Valencia, CA, USA) according to the manufacturer’s instructions. cDNA was synthesized using 5 µg of total RNA, oligo(dT) primers or random primers, and superscript reverse transcriptase (Promega, Madison, WI, USA). The *CaActin* and *NbEF-1α* genes were used as internal controls for RNA quantity in pepper and *N*. *benthamiana*, respectively (all the primers are listed in Supplementary Table S1 at *JXB* online). Individual PCR products were resolved by 1% agarose gel electrophoresis and visualized with ethidium bromide under UV light. Quantitative real-time RT-PCR (qRT-PCR) was performed in a 96-well format using a Light Cycler 480 machine (Roche, Basel, Switzerland).

### Virus-induced gene silencing

A section of 157bp of 3′-untranslated region (UTR) in *CaLecRK-S.5* cDNA was amplified by PCR and cloned into the pTRV2 vector containing part of the *Tobacco rattle virus* (TRV) genome (Ratcliff *et al.*, 2001). The sequence specificity for virus-induced gene silencing (VIGS) was confirmed by BLAST search of genome-wide homology sequence in the CM334 and Zunla database (https://solgenomics.net/tools/blast/?db_id=217). We were unable to find any homologous sequence in other pepper genes. Approximately 1-week-old plants were inoculated with *Agrobacterium tumefaciens* GV3101 carrying TRV-derived plasmids as described previously (Huh *et al.*, 2015). The plants were used approximately 3 weeks after *Agrobacterium* inoculation.

### Sequence alignment and phylogenetic analysis

The full-length protein sequence of CaLecRK-S.5 and its homologs were aligned with ClustalW, and phylogenetic analysis was conducted with Megalign (IntelliGenetics Inc., Mountain View, CA, USA) and MEGA6.

### Trypan blue staining

Dead cells in plant tissues were visualized by trypan blue staining. Infected leaves were stained with trypan blue (10ml phenol, 10ml glycerol, 10ml lactic acid, 10ml water, and 0.02g trypan blue). Infected leaves were boiled for 10min in the staining solution and destained overnight in chloral hydrate.

### Staining for hydrogen peroxide with 3,3′-diaminobenzidine and ROS measurements

Hydrogen peroxide (H_2_O_2_) accumulation was visualized by 3,3′-diaminobenzidine (DAB) staining. Infected leaves were excised and immersed in a 0.1% solution of DAB, and after vacuum infiltration for 20min, the samples were incubated at room temperature for 20h. The stain was discarded and chlorophyll was removed by boiling in 96% ethanol for 10–40min. The brown spots characteristic of the reaction of DAB with H_2_O_2_ were analysed. ROS measurements were performed as described by Asai *et al.* (2008).

### Ion leakage assay

In total, 18 leaf discs (1cm in diameter) per treatment were washed and floated on 5ml of distilled water. Conductivity was measured from 0 to 72h post-inoculation (hpi) using a LAQUAtwin conductivity meter (Horiba Instruments, Kyoto, Japan).

### Confocal laser scanning microscope imaging analysis

Confocal images were collected using a Carl Zeiss LSM 510 META microscope (Carl Zeiss, Oberkochen, Germany). Secondary metabolites were excited with a diode laser (405nm), and emitted light was collected at 450–500nm and 500–530nm. Images were processed using LSM Image Examiner software (Carl Zeiss, Oberkochen, Germany).

### Immunoblot analysis and MAPK assays

Pepper and *N. benthamiana* leaves were frozen in liquid N_2_ and ground to fine powder. Total proteins were extracted as described previously (Huang *et al.*, 2014). For immunoblot analysis, equal amounts of protein were separated by 8 or 15% SDS-PAGE and blotted onto polyvinylidene fluoride membranes. The membranes were probed with monoclonal anti-HA antibody (clone 12CA5; Roche) or polyclonal anti-TMV CP antibody (PVAS-0020; http://knrrb.knrrc.or.kr/index.jsp?rrb=pvgb) at 1:5000 or 1:1000 dilution, respectively. Horseradish peroxidase-conjugated anti-mouse IgG or anti-rabbit IgG (Cell Signaling Technology, Danvers, MA, USA) was used as a secondary antibody for the detection of HA or TMV CP, respectively. MAPK assays were performed as described previously (Huang *et al.*, 2014).

### BABA and wounding treatment

In total, 10mM BABA (Sigma-Aldrich, St Louis, MO, USA) was applied on pepper leaves 12h before TMV-P_0_ infection by spraying (Lazzarato *et al.*, 2009). BABA was dissolved in water, and mock treatment involved water only. Leaves were rubbed twice with a cotton swab and carborundum for wounding treatment.

### *Agrobacterium tumefaciens*-mediated transient overexpression analysis

To generate transient expression constructs, cDNA encoding CaLecRK-S.5 without termination codons was amplified by PCR and subcloned into the binary vector pGWB414 or pBAV154 under the control of the *Cauliflower mosaic virus 35S* promoter or dexamethasone (dex)-inducible promoter, respectively. *CaLecRK-S.5-HA* was expressed in *N. benthamiana* leaves by infiltrating *A. tumefaciens* strain GV3101 carrying the construct (OD_600_ = 0.4).

### RNA-sequencing data analysis

To construct cDNA libraries using the TruSeq RNA library kit, 1 µg of total RNA was used. The protocol consisted of polyA-selected RNA extraction, RNA fragmentation, random hexamer primed reverse transcription, and 100 nt paired-end sequencing with the Illumina HiSeq2000. The libraries were quantified by qRT-PCR. To estimate expression levels and identify alternatively spliced transcripts, the RNA-Seq reads were mapped to the genome of *Capsicum annuum* using TopHat (Trapnell *et al.*, 2009), which is capable of reporting split-read alignments across splice junctions, and were determined using Cufflinks software (Trapnell *et al.*, 2010) in default options. The reference genome sequence of *C*. *annuum* and annotation data were downloaded from The Pepper Genome Platform (PGP) ftp site (http://passport.pepper.snu.ac.kr/?t=PGENOME). The transcript counts at the isoform level were calculated, and the relative transcript abundances were measured in fragments per kilobase of exon per million fragments mapped (FPKM) using Cufflinks. In addition, novel transcripts and alternative splicing transcripts were identified for each sample. These results were obtained using the Cufflinks Reference Annotation Based Transcript Assembly (RABT) method, allowing the discovery of reference transcripts and novel transcripts using the -g option. Raw data were calculated as FPKM of each transcript in each sample by Cufflinks software. We excluded transcripts with >1 zero FPKM values from the total samples. We added 1 to the FPKM value of the filtered transcript to facilitate log_2_ transformation. Filtered data were transformed by logarithm and normalized using the quantile normalization method. For each transcript, fold change was calculated between case and control. Differentially expressed transcripts were determined by adjusting |fold change|≥2 in more than one of the total comparisons. Hierarchical clustering analysis was performed using complete linkage and Euclidean distance as a measure of similarity to display the expression patterns of differentially expressed transcripts satisfying |fold change|≥2 in at least one comparison.

### Gene ontology enrichment analysis

The *C. annuum* genes were annotated with IDs in the *Arabidopsis* database (TAIR9) for enriched gene ontology (GO) term mapping. Singular Enrichment Analysis was performed with FDR = 0.05 using AgriGO (http://bioinfo.cau.edu.cn/agriGO/analysis.php).

## Results

### *Capsicum annuum LecRK-S.5* gene is induced by TMV-P_0_ infection

Previously, we performed DNA microarray experiments to elucidate the molecular mechanism underlying *C. annuum* resistance to TMV-P_0_ infection at the transcription level (Kim *et al.*, 2007). Leaf material used for DNA microarray was derived from plants of the pepper cultivar Bugang, which shows HR upon infection by TMV-P_0_. Compared with mock treatment, among 21 115 genes differentially expressed under TMV-P_0_ infection (Supplementary Table S2), we collected all the 432 expressed sequence tags (ESTs) encoding protein kinases and classified them on the basis of the study of Hanks and Hunter (1995).

PRRs detect PAMPs that are conserved in a wide range of pathogens. Most PRRs are membrane-anchored receptors that contain a TM domain and can thus recognize external stimuli and regulate the early stage of defense responses (Jones and Dangl, 2006; Boller and Felix, 2009). To identify the surface-localized regulators involved in the early stage of defense activation, we further classified 432 ESTs of protein kinases on the basis of the presence of a TM domain (Supplementary Table S3). Among those, eight ESTs of lectin receptor kinases were upregulated during HR against TMV-P_0_ (Supplementary Fig. S1A). To investigate the possible involvement of lectin receptor kinases in pepper disease resistance to TMV-P_0_ infection, we first analysed the induction patterns of seven lectin receptor kinases of eight ESTs by qRT-PCR (Supplementary Fig. S1B). The expression of *PEPPERS0010681* and *PEPPERS0016322* was increased during the resistance response of Bugang to TMV-P_0_, as early as 6h after infection. Induction of the *PEPPERS0010681* transcript was continuous until 48h after infection, but the *PEPPERS0016322* transcript was not accumulated until 48h. Low-level accumulation of transcripts was detected for the other lectin receptor kinase genes. Because HR was fully developed at 48h after TMV-P_0_ inoculation, *PEPPERS0010681* was chosen for further study. To investigate the sequence diversity of the lectin receptor kinase family in pepper, BLAST searches against the pepper genome database (https://solgenomics.net/) were performed using the coding region of *PEPPERS0010681* and lectin receptor kinase genes from Arabidopsis, *N. benthamiana*, tomato (*Solanum lycopersicum*), and potato (*S. tuberosum*). Sequence analysis of the full-length *PEPPERS0010681* clone (CA04g04250) revealed that it encodes a typical L-type lectin receptor kinase (Supplementary Fig. S2B; Bouwmeester and Govers, 2009). To determine the evolutionary relationship among the L-type lectin receptor kinase families, a phylogenetic tree consisting of 18 deduced lectin receptor kinases was generated by the neighbor-joining method using an alignment by ClustalW. CA04g04250 (*PEPPERS0010681* gene) was located in the same clade as SlLecRK-S.5, StLecRK-S.5, NbLecRK-S.5, and AtLecRK-S.5 and was accordingly designated as CaLecRK-S.5. CaLecRK-S.5 shared 90%, 91%, 89%, and 60% amino acid identity at the protein level with SlLecRK-S.5, StLecRK-S.5, NbLecRK-S.5, and AtLecRK-S.5, respectively (Supplementary Fig. S2A). Together, these results suggested that the *PEPPERS0010681* gene encodes an L-type lectin receptor kinase protein, CaLecRK-S.5, that harbors a TM domain like other PRRs and that its transcription is induced by TMV-P_0_ infection.

### TMV-P_0_-mediated resistance responses are compromised in *CaLecRK-S.5*-silenced plants

**Fig. 1.**
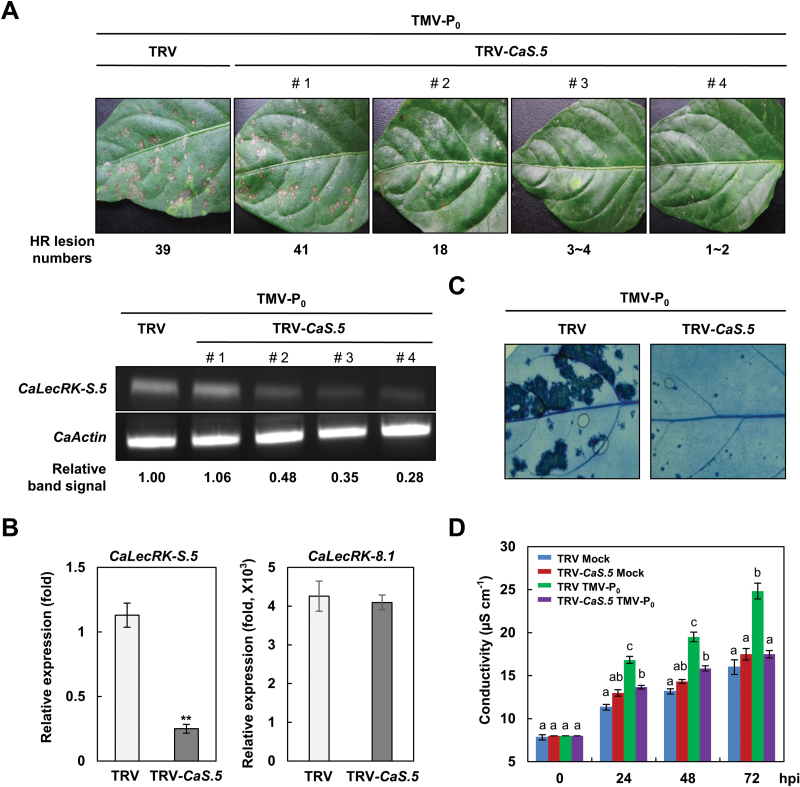
*CaLecRK-S.5*-silenced plants show reduced HR upon TMV-P_0_ infection. *Capsicum annuum* plants with the empty vector (TRV) or those silenced for *CaLecRK-S.5* (TRV-*CaS.5*) were inoculated with TMV-P_0_. (A) Reduced HR lesion numbers in *CaLecRK-S.5* VIGS plants against TMV-P_0_ infection. Photos were taken at 4 d post-inoculation (dpi). RT-PCR analysis of *CaLecRK-S.5* expression was performed 96h post-TMV-P_0_ infection. The intensities of RT-PCR bands were quantified by Multi Gauge V3.0. Expression values were normalized to levels of *CaActin* gene expression. (B) Specific silencing of *CaLecRK-S.5* was monitored by quantitative real-time RT-PCR analysis at 48h post-inoculation (hpi). *CaLecRK-8.1* was used as a control for a close homolog of *CaLecRK-S.5*. Expression values were normalized to levels of *CaActin* gene expression. Data represent means±SD from three independent experiments (Student’s *t*-test, **P<0.05). (C) Reduced cell death response in *CaLecRK-S.5*-silenced pepper leaves is shown by trypan blue staining. Staining was performed at 4 dpi. (D) Ion leakage as an indicator of cell death response was measured. Error bars represent±SD from six biological replicates, and different letters indicate significant differences, as determined by one-way ANOVA, followed by Tukey’s honest significant difference (HSD) test (*P*<0.01).

To investigate the role of the *CaLecRK-S.5* gene in disease resistance of pepper, we reduced the expression level of the *CaLecRK-S.5* gene in *C. annuum* cv. Bugang by VIGS. The VIGS construct was generated by cloning the 3′-UTR of *CaLecRK-S.5* into a TRV vector (Ratcliff *et al.*, 2001). The number of HR lesions upon TMV-P_0_ infection was positively correlated with the *CaLecRK-S.5* transcript level (Fig. 1A). Approximately 52% silencing of *CaLecRK-S.5* (#2) diminished the number of HR lesions to approximately half of that in a TRV control upon TMV-P_0_ infection. Furthermore, 65% and 72% silencing of *CaLecRK-S.5* (#3 and 4) resulted in significantly reduced number of HR lesions. The specific downregulation of *CaLecRK-S.5* by VIGS was demonstrated by the observation that the transcript level of *CaLecRK-8.1*, which shows 53% amino acid similarity with *CaLecRK-S.5*, was not changed between the TRV control and *CaLecRK-S.5*-silenced plants (Fig. 1B). Trypan blue staining of leaves further showed that *CaLecRK-S.5* silencing compromised HR-like cell death in the resistance response to TMV-P_0_ infection (Fig. 1C). In addition, the silenced plants showed significantly lower ion leakage than TRV control plants at 24, 48, and 72h post-inoculation (hpi) (Fig. 1D). Together, these results suggest that the *CaLecRK-S.5* gene is involved in pepper disease resistance to TMV-P_0_ infection.

**Fig. 2.**
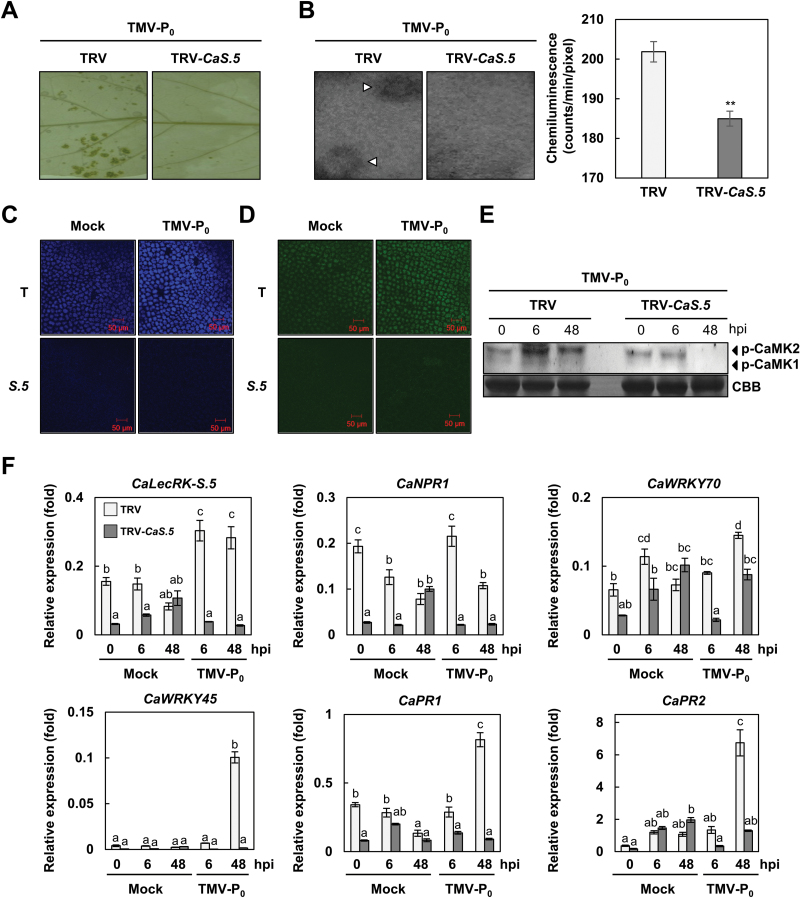
*CaLecRK-S.5* is required for immune responses against TMV-P_0_ infection. *Capsicum annuum* plants with the empty vector (TRV) or those silenced for *CaLecRK-S.5* (TRV-*CaS.5*) were inoculated with TMV-P_0_. Reduced H_2_O_2_ accumulation and ROS burst in *CaLecRK-S.5*-silenced pepper leaves were measured by DAB staining (A) and 0.5mM L-012 solution (B), respectively. Both experiments were conducted at 4 dpi. (B) ROS burst was quantified using a CCD camera and a program equipped with a photon image processor. Data represent means±SD from three independent experiments (Student’s *t*-test, **P<0.05). (C, D) Reduction of secondary metabolite production in *CaLecRK-S.5*-silenced plants. Autofluorescence was observed using a microscope under UV excitation, and emission windows of 420–480nm (C) or 505–530nm (D) were used to capture the signals of secondary metabolites. T denotes TRV control and *S.5* for TRV-*CaLecRK-S.5*. (E) Activity of MAPK phosphorylation was detected using anti-phospho-p44/42 MAPK antibody. Coomassie Brilliant Blue (CBB) staining indicates equal loading. (F) Total RNA was extracted from *C. annuum* plants with the empty vector (TRV) or those silenced for *CaLecRK-S.5* (TRV-*CaS.5*) 0, 6 and 48h after mock or TMV-P_0_ treatment. Relative expression levels of *CaLecRK-S.5*, *CaNPR1*, *CaWRKY70*, *CaWRKY45*, *CaPR1*, and *CaPR2* were analysed by quantitative real-time RT-PCR. Expression values were normalized to levels of *CaActin* gene expression. Error bars represent ±SD from three biological replicates, and different letters indicate significant differences, as determined by one-way ANOVA, followed by Tukey’s HSD test (*P*<0.05).

Given that the suppression of *CaLecRK-S.5* expression compromised pepper disease resistance to TMV-P_0_, we further analysed the physiological and molecular mechanisms involved in the reduced HR in *CaLecRK-S.5*-silenced plants. The accumulation of H_2_O_2_, as detected by 3,3′-diaminobenzidine (DAB) staining, was induced by TMV-P_0_ infection in the leaves of TRV control plants. However, significantly reduced H_2_O_2_ was detected in the leaves of *CaLecRK-S.5*-silenced plants (Fig. 2A). Moreover, the leaves of *CaLecRK-S.5*-silenced plants showed diminished intensity in the areas stained by the chemiluminescence probe L-012, which detects ROS bursts (Fig. 2B). These observations indicate that *CaLecRK-S.5* is involved in the generation or accumulation of H_2_O_2_ upon TMV-P_0_ infection.

One of the key mechanisms underlying pathogen resistance is the temporal and spatial accumulation of secondary metabolites (Hartmann, 2007; Bednarek, 2012; Pusztahelyi *et al.*, 2015). The accumulation of secondary metabolites can be detected by microscopy without any treatment (Talamond *et al.*, 2015). Autofluorescence may be observed by confocal laser scanning microscopy under UV laser. Some phenolic compounds and alkaloids in plants emit in the blue region (450–500nm), while some flavonoids and terpenoids emit in the green region (500–530nm). TMV-P_0_ infection induced secondary metabolites such as phenolic compounds or alkaloids (420–480nm) and flavonoids or terpenoids (505–530nm) in TRV control plants (Fig. 2C, D). Interestingly, compared with TRV control plants, the production of these secondary metabolites was markedly diminished in *CaLecRK-S.5*-silenced plants upon TMV-P_0_ treatment as well as mock treatment.

MAPK activation is also the key component of the defense mechanism upon TMV-P_0_ infection (Liu *et al.*, 2004; Takabatake *et al.*, 2007; Huh *et al.*, 2015). In contrast to TRV control plants, the silencing of *CaLecRK-S.5* failed to trigger TMV-P_0_ infection-mediated phosphorylation of CaMK1 and CaMK2, orthologs of Arabidopsis mitogen-activated protein kinase (MPK) 3 and MPK6, respectively (Fig. 2E). As expected, TMV-P_0_ infection triggered CaMK1 and CaMK2 phosphorylation in TRV control plants. According to previous hot pepper microarray analysis (Kim *et al.*, 2007), TMV-P_0_ infection leads to transcriptional reprogramming in pepper leaves during HR. To investigate whether *CaLecRK-S.5* silencing alters the expression of defense-related genes during TMV-P_0_ infection, transcript levels of *CaNPR1*, *CaWRKY70*, *CaWRKY45*, *CaPR1*, and *CaPR2* at 6 and 48h after mock or TMV-P_0_ treatment were measured by qRT-PCR (Fig. 2F). *CaLecRK-S.5*-silenced plants showed significantly attenuated induction patterns of *CaNPR1*, *CaWRKY70*, *CaWRKY45*, *CaPR1*, and *CaPR2* during TMV-P_0_ infection. Interestingly, in response to mock treatment, the expression of *CaNPR1*, which plays a crucial role in immunity including SAR, was markedly lower in *CaLecRK-S.5*-silenced plants than in TRV control plants. Taken together, these results indicate that the expression of defense-related genes and the transcript level of *CaLecRK-S.5* are highly correlated. Thus, all results together suggest that *CaLecRK-S.5* positively regulates the activation of immune responses upon TMV-P_0_ infection.

### *CaLecRK-S.5* is associated with broad-spectrum resistance to plant pathogens

**Fig. 3.**
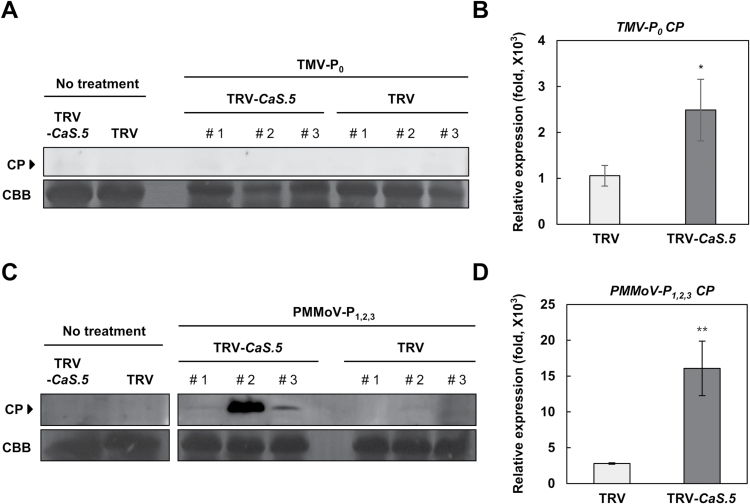
*CaLecRK-S.5* is involved in basal resistance to tobamovirus infection. Total protein or RNA was extracted from upper uninoculated leaves of TRV control and *CaLecRK-S.5*-silenced plants 14 d after TMV-P_0_ (A, B) and 9 d after PMMoV-P_1,2,3_ (C, D) infection, respectively. Spreading of virus was assessed by western blot analyses or qRT-PCR assay using polyclonal anti-TMV CP antibody or specific primers for each viral CP gene. Coomassie Brilliant Blue staining indicates equal loading for western blot analyses. Expression values of qRT-PCR were normalized to levels of *CaActin* gene expression. Data represent means±SD from three independent experiments (Student’s *t*-test, *P<0.1, **P<0.05). The numbers on immunoblots indicate individual samples.

To investigate whether *CaLecRK-S.5* is indeed involved in TMV-P_0_ resistance, TRV vector control and *CaLecRK-S.5*-silenced pepper plants were infected with TMV-P_0_ and tested for the presence of TMV-P_0_ coat protein (CP) and viral RNA in upper uninoculated leaves (Fig. 3A, B). However, negligible TMV-P_0_ CP increase was detected in either *CaLecRK-S.5*-silenced or TRV control plants even 14 dpi by western blot analysis (Fig. 3A). In contrast, compared with TRV control plants, there was a clear increase in the accumulation of viral RNA in upper uninoculated leaves of *CaLecRK-S.*5-silenced plants (Fig. 3B). In *C. annuum* L. cv. Bugang, the resistance conferred by the *L*^*2*^ gene is initiated by the recognition of CP and effective against TMV-P_0_, but PMMoV-P_1,2,3_ escapes from the *L*^*2*^ gene-mediated resistance and can therefore spread systemically (de la Cruz *et al.*, 1997; Antignus *et al.*, 2008). We then postulated that *CaLecRK-S.5* is not directly associated with the function of *L*^*2*^ in virus resistance response. To investigate the relationship between *CaLecRK-S.5* and *L*^*2*^, we inoculated TRV control and *CaLecRK-S.5*-silenced plants with PMMoV-P_1,2,3_ (Fig. 3C, D). CP of PMMoV-P_1,2,3_ was detected in upper uninoculated leaves of *CaLecRK-S.5*-silenced plants as early as 9 dpi, but much smaller amounts of CP were detected in TRV control plants at that time (Fig. 3C). Moreover, the accumulation of PMMoV-P_1,2,3_ viral RNA in upper uninoculated leaves was approximately five times greater in *CaLecRK-S.5*-silenced plants than in TRV control plants (Fig. 3D). Further, *CaLecRK-S.5* was induced by PMMoV-P_1,2,3_ infection, as in the case of TMV-P_0_ infection (Supplementary Fig. S3). These results indicate that *CaLecRK-S.5* is involved in basal resistance against tobamovirus infection but not directly associated with the function of the *L*^*2*^ gene *per se*.

**Fig. 4.**
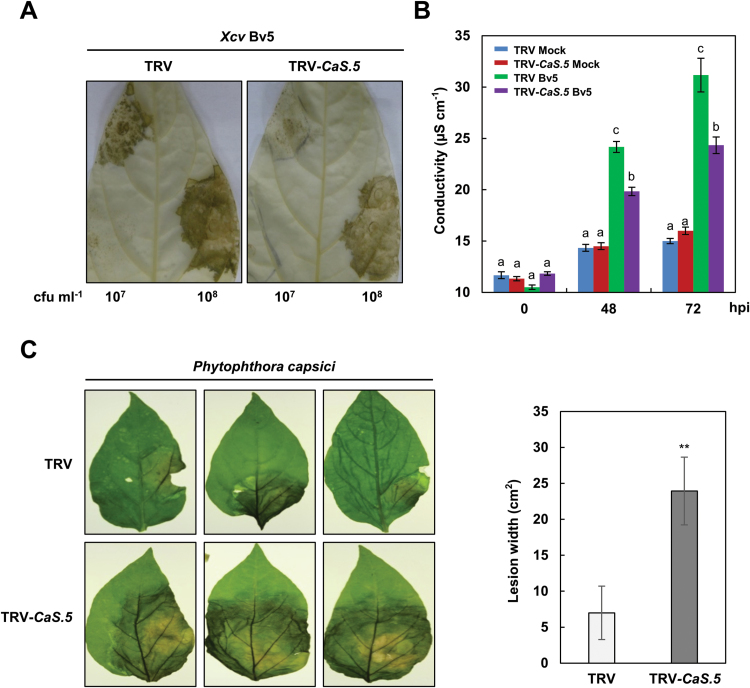
*CaLecRK-S.5* is associated with broad-spectrum resistance in *C. annuum* plants. (A) Reduced HR-like cell death of *CaLecRK-S.5* VIGS plants to *Xcv* Bv5 infection. Photographs were taken 7 dpi. Ethanol was used to remove chlorophyll. (B) Ion leakage was measured upon *Xcv* Bv5 treatment in comparison with control. Error bars represent ±SD from six biological replicates, and different letters indicate significant differences, as determined by one-way ANOVA, followed by Tukey’s HSD test (*P*<0.01). (C) *CaLecRK-S.5* VIGS plants showed higher susceptibility to *Phytophthora capsici*. TRV control and *CaLecRK-S.5*-silenced plants were inoculated with *P. capsici* spores. Photographs were taken and lesion widths were measured 5 dpi. Data are means±SD from six replicates (Student’s *t*-test, **P<0.05).

To determine whether *CaLecRK-S.5* confers broad-spectrum resistance to other pathogens in addition to tobamovirus, the bacterial pathogen *Xcv*, the causal agent of bacterial spot disease in pepper plants, and *P. capsici*, an oomycete pathogen that causes blight and fruit rot of peppers, were inoculated on TRV vector control and *CaLecRK-S.5*-silenced pepper plants (Fig. 4). Inoculation with the *Xcv* avirulent strain Bv5-4a (*Xcv* Bv5) induces HR-like cell death accompanied by ROS accumulation and defense-related gene expression in pepper leaves (Lee and Hwang, 2005). Reduced HR-like cell death indicates attenuation of disease resistance to *Xcv* Bv5 infection (Choi *et al.*, 2012). Compared with TRV control plants, *CaLecRK-S.5*-silenced plants inoculated with *Xcv* Bv5 (10^7^ and 10^8^ cfu ml^−1^) showed significantly reduced HR-like cell death (Fig. 4A) and approximately 20% diminished conductivity at 48 and 72h after *Xcv* Bv5 inoculation (10^7^ cfu ml^−1^), respectively (Fig. 4B). *CaLecRK-S.5*-silenced plants were also challenged with *P. capsici* to test the involvement of *CaLecRK-S.5* in resistance to oomycete pathogens. The silencing of *CaLecRK-S.5* conferred higher susceptibility to *P. capsici* infection than the TRV control. The lesion width of *CaLecRK-S.5*-silenced plants was approximately four times larger than that of TRV control plants (Fig. 4C). Taken together, these results suggest that *CaLecRK-S.5*-mediated defense responses contribute to broad-spectrum resistance.

### Priming is a major factor in *CaLecRK-S.5*-mediated defense response

**Fig. 5.**
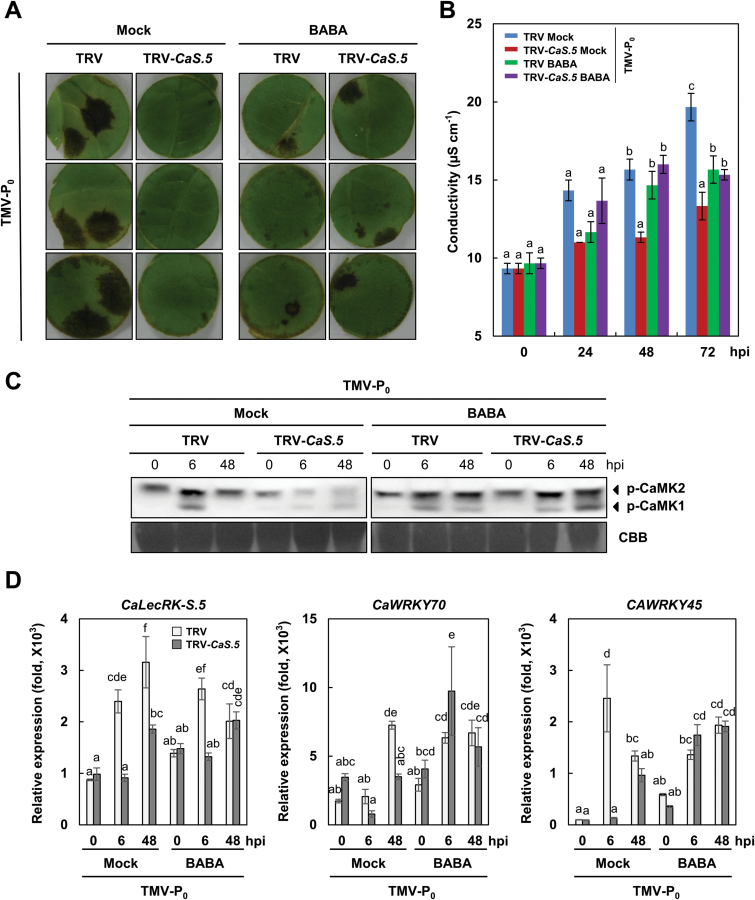
BABA-induced priming restores the defense responses in *CaLecRK-S.5*-silenced plants upon TMV-P_0_ infection. *Capsicum annuum* plants with the empty vector (TRV) or those silenced for *CaLecRK-S.5* (TRV-*CaS.5*) were treated with mock or 10mM BABA by spraying 12h before TMV-P_0_ infection. (A) HR was observed 4 d after TMV-P_0_ infection. These results show three typical replicates of nine independent ones. (B) Ion leakage was measured 0, 24, 48, and 72h after TMV-P_0_ infection. Error bars represent ±SD from six biological replicates, and different letters indicate significant differences, as determined by one-way ANOVA, followed by Tukey’s HSD test (*P*<0.05). (C) Suppression of MAPK phosphorylation by *CaLecRK-S.5* silencing upon TMV-P_0_ treatment was restored by BABA-mediated priming. Phosphorylation of MAPK was detected with anti-phospho-p44/42 MAPK antibody. Coomassie Brilliant Blue staining indicates equal loading. (D) Relative expression levels of *CaLecRK-S.5*, *CaWRKY70*, and *CaWRKY45* were analysed by quantitative real-time RT-PCR. Expression values were normalized to levels of *CaActin* gene expression. Error bars represent ±SD from three biological replicates, and different letters indicate significant differences, as determined by one-way ANOVA, followed by Tukey’s HSD test (*P*<0.05).

Priming potentiates defense responses to a broad spectrum of plant pathogens (Conrath *et al.*, 2006; Ahmad *et al.*, 2010). ROS and MAPK play a crucial role in priming (Canton and Grinstein, 2014; Conrath *et al.*, 2015). β-Aminobutyric acid (BABA) is a well-known chemical agent that triggers priming (Jakab *et al.*, 2001). In this study, we showed that ROS burst and MAPK activation are involved in *CaLecRK-S.5*-mediated resistance, which confers broad-spectrum resistance to viral, bacterial, and oomycete pathogens (Figs 2, 3 and 4). To investigate whether priming is involved in the mechanism underlying *CaLecRK-S.5*-mediated resistance, 10mM BABA was applied to TRV control and *CaLecRK-S.5*-silenced plants 12h before TMV-P_0_ infection (Fig. 5). The weak HR induced upon TMV-P_0_ infection in *CaLecRK-S.5*-silenced plants was completely rescued to the level of the TRV control plants by pretreatment with BABA (Fig. 5A). It was previously shown that plants pretreated with BABA before TMV infection showed reduced HR lesion size and numbers than untreated plants (Lazzarato *et al.*, 2009; Fig. 5A). Recovery of the HR phenotype by BABA pretreatment was quantified by ion leakage measurement (Fig. 5B). TMV-P_0_-mediated ion leakage in BABA-pretreated *CaLecRK-S.5*-silenced plants was comparable to that in BABA-pretreated TRV control plants. TMV-P_0_-mediated ion leakage in BABA-pretreated TRV control and *CaLecRK-S.5*-silenced plants was lower than that in untreated TRV control plants but higher than that in untreated *CaLecRK-S.5*-silenced plants. The abolition of MAPK activation and reduction of defense-related gene expression in *CaLecRK-S.5*-silenced plants in response to TMV-P_0_ infection were also restored by BABA pretreatment (Fig. 5C, D). Compared with BABA-untreated TRV control plants, the accumulation of phosphorylated MAPK upon TMV-P_0_ infection was significantly reduced in BABA-untreated *CaLecRK-S.5*-silenced plants, but BABA pretreatment rescued MAPK activation in *CaLecRK-S.5*-silenced plants (Fig. 5C). The reduction of defense-related gene expression in *CaLecRK-S.5*-silenced plants was also rescued by BABA pretreatment (Fig. 5D). Reduced expression of *CaWRKY70* and *CaWRKY45* in *CaLecRK-S.5*-silenced plants at 6 hpi was completely restored by BABA pretreatment. In fact, the silencing of *CaLecRK-S.5* was maintained until 6 hpi, but the silencing of *CaLecRK-S.5* was abolished at 48 hpi under BABA pretreatment conditions. This result can probably be explained by a previous study showing that BABA pretreatment before TMV infection induces reduced HR size and numbers (Fig. 5A, B; Lazzarato *et al.*, 2009). Together, these results indicate that priming is a key factor in *CaLecRK-S.5*-mediated defense response.

 Wounding treatment is known to induce plant priming, leading to faster and stronger immunity to *B. cinerea* (Chassot *et al.*, 2008). Wounded leaves produce ROS and activate MAPK, which play crucial roles in resistance to *B. cinerea* (Seo *et al.*, 2007; Ren *et al.*, 2008; Beneloujaephajri *et al.*, 2013). To further confirm the role of priming in *CaLecRK-S.5*-mediated resistance, wounding stress was applied by rubbing leaves with carborundum and a cotton swab. In pepper, expression of the *CaLecRK-S.5* gene was induced by wounding stress (Supplementary Fig. S4A). *CaPin2* was used as a wounding marker gene. *Nicotiana benthamiana* leaves infiltrated with *Agrobacterium tumefaciens* expressing *CaLecRK-S.5-HA* under the control of the CaMV *35S* promoter (*35S*::*CaS.5-HA*) (Supplementary Fig. S4B) were applied with wounding stress treatment 24h after *Agrobacterium* infiltration (Supplementary Fig. S4C, D). Transient overexpression of *CaLecRK-S.5 per se* did not trigger MAPK activation or ROS burst without stimulation. However, wound-induced MAPK activation and ROS burst were much stronger in *35S*::*CaS.5-HA* leaves than in empty vector control leaves.

To investigate whether the overexpression of *CaLecRK-S.5* triggers the accumulation of defense signaling components, *N. bethamiana* leaves were infiltrated with *A. tumefaciens* carrying *CaLecRK-S.5-HA*, whose transcriptional activation is under the control of a dexamethasone (dex)-inducible promoter (*dex*::*CaS.5-HA*). In total, 30 µM dex was applied 24h after *Agrobacterium* infiltration for induction. CaLecRK-S.5-HA was detected by a western blot assay at 6h after dex treatment, and its expression was diminished at 24h after dex treatment (Supplementary Fig. S5A). Transient overexpression of *CaLecRK-S.5* stimulated the expression of defense-related genes, *NbPR4b*, *SAR8.2*, and *NbRbohB* (Supplementary Fig. S5B). These results, together with the rescue of HR by BABA pretreatment, suggest that priming is a critical component of the function of the *CaLecRK-S.5* gene.

### SAR is abolished in *CaLecRK-S.5*-silenced plants

**Fig. 6.**
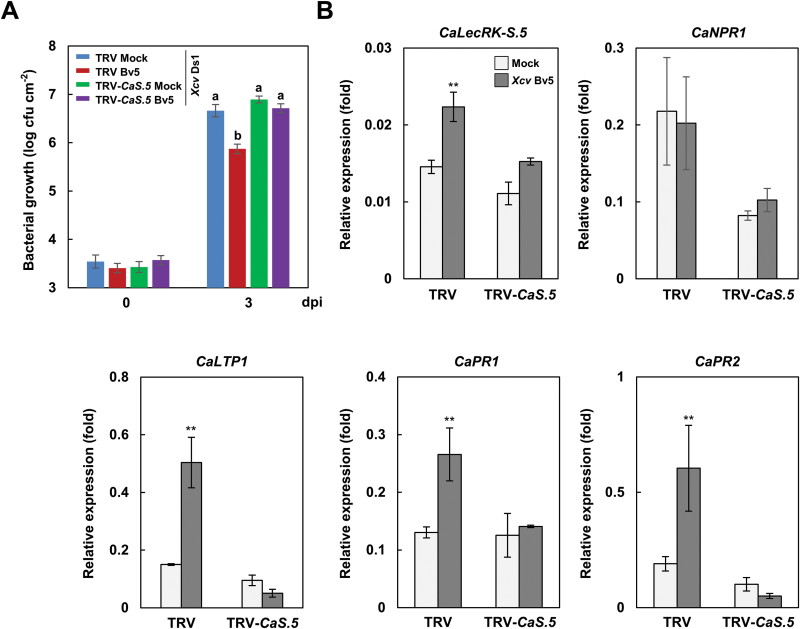
*CaLecRK-S.5* is critical for systemic acquired resistance (SAR) to *Xcv* infection. (A) Bacterial growth was analysed from TRV control and *CaLecRK-S.5*-silenced plants 0 or 3 dpi with virulent *Xcv* Ds1 (10^6^ cfu ml^−1^). *Xcv* Ds1 was infiltrated in upper leaves after 2 d of primary leaf treatment with mock or *Xcv* Bv5 (10^7^ cfu ml^−1^) to test SAR. Error bars represent ±SD from 10 independent replicates, and different letters indicate significant differences, as determined by one-way ANOVA, followed by Tukey’s HSD test (*P*<0.05). (B) Compared with TRV control plants, the expression of SAR-induced marker genes was diminished in *CaLecRK-S.5*-silenced plants. Total RNA was extracted from distal upper uninoculated leaves 48h after inoculation of the primary leaf with mock or avirulent *Xcv* Bv5 (10^7^ cfu ml^−1^). Relative expression levels of *CaLecRK-S.5*, *CaNPR1*, *CaLTP1*, *CaPR1*, and *CaPR2* were analysed by qRT-PCR. Expression values were normalized to levels of *CaActin* gene expression. Data represent the means±SD from three independent experiments (Student’s *t*-test, **P<0.05).

SAR is a defense priming state that is induced by local infection and confers broad-spectrum resistance to further pathogen challenge in unchallenged tissue (Conrath *et al.*, 2015). To investigate the impact of impaired *CaLecRK-S.5* gene expression on SAR, we examined the propagation of the *Xcv* virulent strain Ds1 (*Xcv* Ds1) in systemic leaves of TRV control and *CaLecRK-S.5*-silenced plants 2 d after the inoculation of primary leaves with the *Xcv* avirulent strain Bv5 (Fig. 6A). Notably, SAR was not induced in *CaLecRK-S.5*-silenced plants but was significantly induced in TRV control plants. Thus, *Xcv* Bv5-treated TRV control plants harbored approximately 10 times lower *Xcv* Ds1 than mock-treated TRV control plants. In contrast, *CaLecRK-S.5*-silenced plants showed no difference in the *Xcv* Ds1 population between mock- and *Xcv* Bv5-treated plants.

To further investigate a potential role of *CaLecRK-S.5* in SAR, we monitored the transcript levels of *CaLecRK-S.5*, *CaNPR1*, *CaLTP1*, *CaPR1*, and *CaPR2* in systemic leaves of TRV control and *CaLecRK-S.5*-silenced plants 2 d after *Xcv* Bv5 infiltration of primary leaves (Fig. 6B). The marker genes were upregulated by SAR in systemic leaves of TRV control plants, but these inductions were diminished in *CaLecRK-S.5*-silenced plants. In particular, *CaNPR1* was not induced by SAR in TRV control plants, but *CaLecRK-S.5*-silenced plants showed significantly reduced expression of *CaNPR1* in both non-SAR and SAR conditions. NPR1 plays a critical role in SAR (Mou *et al.*, 2003). In an uninduced state, NPR1 is present as an oligomeric form in the cytoplasm. Upon SAR induction, accumulation of SA triggers a change in cellular reduction potential, resulting in reduction of NPR1 to a monomeric form. Monomeric NPR1 translocates to the nucleus where it functions as a coactivator of gene transcription. These results indicate that there is sufficient CaNPR1 in TRV control plants to function in SAR as a monomeric form even though the transcript level is not induced by SAR; however, the amount of CaNPR1 is too small to function in *CaLecRK-S.5*-silenced plants and therefore SAR is abolished in the silenced plants. These observations together suggest that *CaLecRK-S.5* plays a positive role in SAR.

### Transcriptome profiling of *CaLecRK-S.5*-silenced plants in response to TMV-P_0_ infection

**Fig. 7.**
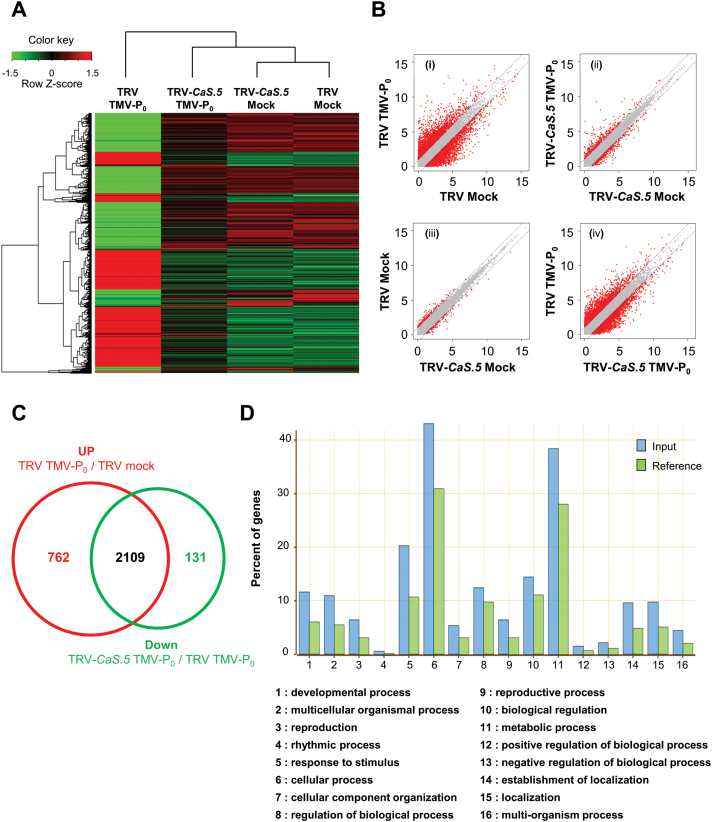
Transcriptome analysis reveals that expression of numerous transcripts, particularly those involved in defense and priming mechanisms, was changed in *CaLecRK-S.5*-silenced plants. RNA sequencing analysis of genes from empty vector (TRV) or *CaLecRK-S.5* VIGS (TRV-*CaS.5*) plants 24h after mock or TMV-P_0_ treatment. (A) Hierarchical clustering of gene expression. Fold-change values (log_2_ based) of 6403 genes were colored red for upregulation and green for downregulation, as illustrated in the color key. (B) Scatter plot of gene expression between two selected test groups. (i) TRV plants with TMV-P_0_ treatment/TRV plants with mock treatment. (ii) TRV-*CaS.5* plants with TMV-P_0_ treatment/TRV-*CaS.5* plants with mock treatment. (iii) TRV plants with mock treatment/TRV-*CaS.5* plants with mock treatment. (iv) TRV plants with TMV-P_0_ treatment/TRV-*CaS.5* plants with TMV-P_0_ treatment. Red dots show significantly altered genes (|fold change|≥2). Gray line indicates two-fold change. (C) Venn diagram shows the number of upregulated genes on TMV-P_0_ infection (compared with mock) in TRV plants and downregulated genes by *CaLecRK-S.5* silencing (compared with TRV) under TMV-P_0_-infected conditions. (D) The 2109 overlapping genes were annotated with IDs in the Arabidopsis database (TAIR9) for enriched gene ontology (GO) term mapping (Agrigo). Blue and green colors indicate input and reference, respectively. *Y*-axis: percentage of genes, *X*-axis: GO annotation.

To gain further insight into the molecular resistance mechanism mediated by *CaLecRK-S.5*, TRV control and *CaLecRK-S.5*-silenced plants were treated with mock or TMV-P_0_, and total RNA was extracted 24h after treatment for Illumina RNA sequencing (Fig. 7). A minimum length of 36bp for sequencing reads was used to identify *C. annuum* genes. The Illumina sequencing reads were mapped to the reference genomic DNA sequence of *C. annuum* (https://solgenomics.net/), and Illumina sequencing was performed twice for more accurate analysis. The overall read-mapping proportions were 73.1%, 70.7%, 75.4%, and 78.6% for mock-treated TRV control, TMV-treated TRV control, mock-treated *CaLecRK-S.5*-silenced, and TMV-treated *CaLecRK-S.5*-silenced plants, respectively. Among 30 242 total transcripts, the expression of 15 749 *C. annuum* transcripts was detected in at least one sample. Of these, 6403 transcripts showed significant changes (|fold change|≥2) in gene expression (Supplementary Table S4 and Fig. 7A). In TRV control plants, compared with mock treatment, 2871 and 3002 genes were significantly upregulated and downregulated in response to TMV-P_0_, whereas 378 and 357 genes were upregulated and downregulated, respectively, in *CaLecRK-S.5*-silenced plants (Fig. 7B). Compared with mock-treated *CaLecRK-S.5*-silenced plants, 143 and 118 genes were upregulated and downregulated in mock-treated TRV control plants, whereas compared with TMV-P_0_-treated *CaLecRK-S.5*-silenced plants, 2240 and 2285 genes were upregulated and downregulated in TMV-P_0_-treated TRV control plants. Compared with mock treatment, the transcripts upregulated by TMV-P_0_ treatment in TRV control plants (2871 genes) but downregulated by *CaLecRK-S.5* silencing in response to TMV-P_0_ infection (2240 genes) may be involved in *CaLecRK-S.5*-mediated resistance response (Fig. 7C and Supplementary Table S5). Gene ontology (GO) term enrichment analysis showed that ‘response to stimulus’, ‘cellular process’, and ‘metabolic process’ were significantly enriched in the 2109 overlapping genes (Fig. 7D). The GO terms ‘response to hormone stimulus’, ‘defense response’, ‘response to temperature stimulus’, and ‘response to oxidative stress’ were overrepresented in the GO ‘response to stimulus’ category (Supplementary Fig. S6A). Similarly, the GO terms ‘phosphorylation’, ‘carboxylic acid metabolic process’, ‘cellular nitrogen compound metabolic process’, ‘cellular carbohydrate metabolic process’, ‘lipid metabolic process’, ‘protein modification process’, ‘organic acid biosynthetic process’, and ‘cellular amino acid and derivative metabolic process’ were overrepresented in the GO ‘cellular process’ and ‘metabolic process’ categories (Supplementary Fig. S6B). Interestingly, genes such as *PATHOGENESIS-RELATED GENE 1* (*PR1*), *PATHOGENESIS-RELATED GENE 3* (*PR3*), *PATHOGENESIS-RELATED GENE 4* (*PR4*), *HEAT SHOCK PROTEIN 17* (*HSP17*), *HEAT SHOCK PROTEIN 70* (*HSP70*), *HEAT SHOCK PROTEIN 90.1* (*HSP90.1*), *LIPID TRANSFER PROTEIN 1* (*LTP1*), *LIPID TRANSFER PROTEIN 2* (*LTP2*), *LIPID TRANSFER PROTEIN 12* (*LTP12*), *LIPOXYGENASE 2* (*LOX2*), and *CYTOCHROME P450 FAMILY 83 SUBFAMILY B POLYPEPTIDE 1* (*CYP83B1*), which are associated with plant immunity and SAR, were downregulated in mock-treated *CaLecRK-S.5*-silenced plants in comparison with mock-treated TRV control plants (Supplementary Table S6). These results indicate that some immunity- and priming (or SAR)-related genes were downregulated in *CaLecRK-S.5*-silenced plants with no pathogen infection.

## Discussion

The first layer of defense against infection by other organisms is the innate immune system. One of the emerging examples of inducible lectin receptor kinases with a role as PRR in the immune response is a lectin S-domain receptor kinase, which mediates lipopolysaccharide sensing in Arabidopsis (Ranf *et al.*, 2015). In the present study, we identified a large number of pepper *lectin receptor kinase* genes by analysis of a transcriptome database in response to TMV-P_0_ infection (Supplementary Table S3 and Supplementary Fig. S1). Among them, we demonstrated the link between a *CaLecRK-S.5* gene and the basal defense response by biochemical, microscopic, and gene expression analysis and functional studies.

*CaLecRK-S.5* silencing resulted in compromised resistance against viruses such as TMV-P_0_ and PMMoV-P_1,2,3_, the bacterial pathogen *Xcv* Bv5, and the oomycete *P. capsici* (Figs 2–4). In response to TMV-P_0_ infection, *CaLecRK-S.5*-silenced plants showed diminished TMV-P_0_-mediated defense responses such as HR, ROS burst, and MAPK activation compared with TRV control plants (Figs 1 and 2). Viral RNA was accumulated to higher levels in upper uninoculated leaves of *CaLecRK-S.5*-silenced plants than in those of TRV control plants. However, no TMV-P_0_ CP was detected by the western blot assay in either plant (Fig. 3A, B). In contrast, both CP and RNA of virulent strain PMMoV-P_1,2,3_ were detected at higher levels in *CaLecRK-S.5*-silenced plants than in TRV control plants (Fig. 3C, D). These results suggest that *CaLecRK-S.5* is associated with basal defense responses. Thus, although some TMV-P_0_ RNA can move to upper uninoculated leaves of *CaLecRK-S.5*-silenced plants as basal defense is abolished, because the expression of the *L*^*2*^ gene, which recognizes CP and causes the inhibition of viral amplification, was maintained, we were unable to detect TMV-P_0_ CP at the protein level. However, we verified that increased susceptibility to TMV-P_0_ in *CaLecRK-S.5*-silenced plants is correlated with decrease of ROS burst, secondary metabolite accumulation, CaMK1 and CaMK2 phosphorylation, and defense-related gene expression, which are involved in *L*^*2*^ gene function (Figs 2 and 7). Taken together, these results indicate that *CaLecRK-S.5*-mediated basal defense is critical for the potentiation of *L*^*2*^ gene-mediated defense responses.

Priming is the preconditioned state of a plant that is induced by PAMPs, DAMPs, herbivore-associated molecular patterns (HAMPs), effectors, or chemicals and causes faster and stronger defense mechanisms against subsequent pathogen attack than against initial pathogen attack. Priming in plants confers broad-spectrum resistance with minimal consumption of energy for growth and reproduction. Priming is thus considered to be a cost-efficient resistance strategy in pathogen-containing environments (Ahmad *et al.*, 2010; Conrath *et al.*, 2015).

Transcriptome profiling analysis showed that the expression of genes categorized into primary metabolism, such as ‘cellular amino acid and derivative metabolic process’, ‘carboxylic acid metabolic process’, and ‘carbohydrate metabolic process’, was significantly reduced (fold change ≤−2) in a *CaLecRK-S.5*-silenced plant responding to TMV-P_0_ infection (Fig. 7 and Supplementary Fig. S6). The preinvasion stage of priming targets the primary metabolism of carbohydrate, carboxylic acids, and amino acids (Gamir *et al.*, 2014). BABA pretreatment increases the accumulation of indole-3-carboxylic acid, possibly triggering accelerated callose accumulation in a resistance response to the necrotrophic pathogen *Plectosphaerella cucumerina* (Gamir *et al.*, 2012). Azelaic acid, a nine-carbon dicarboxylic acid, protects Arabidopsis from the pathogen *Pseudomonas syringae* by priming, overaccumulating SA, and inducing *PR1* expression. The carbohydrate derivative glycerol-3-phosphate (G3P) is an essential signal mediating SAR by connecting AZA with lipid transfer proteins (LTPs) such as DIR1 and AZI1 (Jung *et al.*, 2009; Yu *et al.*, 2013). Carbohydrates further contribute to priming by producing inactive sugar conjugates during the pre-invasion priming stage and by being rapidly converted into the free active form in the pathogen invasion stage (Gamir *et al.*, 2014). The status of amino acid metabolism also affects plant resistance to pathogens. Stuttmann *et al.* (2011) reported that mutations in *dihydrodipicolinate synthase 2* and *aspartate kinase 2* genes led to increased resistance against the oomycete *Hyaloperonospora arabidopsidis* (*Hpa*) via the overaccumulation of Asp-derived amino acids Met, Thr, and Ile. Among them, pretreatment with Thr suppressed *Hpa*, indicating that Thr is a key amino acid in this priming effect. Another key amino acid is Trp, given that camalexin, a Trp-derived secondary metabolite, is involved in basal and induced resistance to *B. cinerea* (Ferrari *et al.*, 2003; Kliebenstein *et al.*, 2005; Chassot *et al.*, 2008). These results suggest that *CaLecRK-S.5* plays a positive role in priming by controlling primary metabolism.

In response to mock treatment, the expression levels of *CaHSP70*, *CaHSP90*, and *CaLTPs* in a *CaLecRK-S.5*-silenced plant were significantly diminished in comparison with those in a TRV control plant (Supplementary Table S6). The expression of *CaLTP1* was particularly decreased in *CaLecRK-S.5*-silenced plants during SAR (Fig. 6B). HSP90 forms a molecular chaperone complex that activates cytosolic R proteins and thus mediates plant immunity (Kadota and Shirasu, 2012). HSP70 interacts with the co-chaperone SGT1 and is required for *P. infestans* elicitin INF1-mediated HR (Kanzaki *et al.*, 2003). In addition, CaHSP70 confers resistance to *Xcv* by inducing HR (Kim and Hwang, 2015). A *CaLecRK-S.5*-silenced plant showed the suppression of HR and higher susceptibility to TMV, *Xcv*, and *P. capsici*, suggesting that CaHSP70 is involved in *CaLecRK-S.5*-mediated resistance (Fig. 4). Both HSPs are upregulated by SAR (by *P. syringae* pv. *maculicola* ES4326) and S-methyl-1,2,3-benzothiadiazole-7-carbothioate (BTH), considered to be a functional SA analog (Gruner *et al.*, 2013).

Interestingly, the expression of *CaNPR1* was also lower in *CaLecRK-S.5*-silenced plants than in TRV control plants (Figs 2F and 6B). NPR1 functions in an SA-dependent defense response and SAR by triggering the induction of *PR* genes and genes associated with the secretory pathway (Mou *et al.*, 2003; Wang *et al.*, 2005). Both *P. fluorescens* WCS417r-induced ISR and BABA-mediated priming protect plants from some overlapping pathogens, indicating that they share similar resistance responses, even though WCS417r-induced ISR primes the induction of JA-related genes and BABA primes the induction of SA-related genes (Zimmerli *et al.*, 2000; Van der Ent *et al.*, 2009). NPR1 not only regulates WCS417r-induced ISR but also plays a major role in BABA-mediated induction of transcriptional factor genes such as *WRKY70*, indicating its important role in regulating and connecting different priming pathways (Van der Ent *et al.*, 2009). Taken together, these results suggest that *CaLecRK-S.5* is involved in complex and diverse priming mechanisms.

BABA-mediated priming and resistance are altered in the *lecrk-VI.2* mutant (Singh *et al.*, 2012). In contrast to the *lecrk-VI.2* mutant, priming by BABA treatment restored defense responses in *CaLecRK-S.5*-silenced plants upon TMV-P_0_ infection (Fig. 5). The levels of HR, MAPK activation, and marker gene expression were restored to the levels of TRV control by BABA pretreatment in *CaLecRK-S.5*-silenced plants. These results indicate that *CaLecRK-S.5* and *LecRK-VI.2* function as positive regulators in plant immunity through priming, but via different pathways. *CaLecRK-S.5* is not involved in the perception of BABA.

*NPR1* overexpression in Arabidopsis results in constitutive priming and broad-spectrum resistance (Friedrich *et al.*, 2001). SA or BTH treatment causes increased levels of PRRs such as FLAGELLIN SENSING2 (FLS2), BRASSINOSTEROID INSENSITIVE1-ASSOCIATED RECEPTOR KINASE1 (BAK1), and CHITIN ELICITOR RECEPTOR KINASE1 (CERK1) in the microsomal membrane fraction and thus potentiates the responsiveness of plants to PAMPs (Tateda *et al.*, 2014). In fact, *BAK1* overexpression in Arabidopsis led to increased resistance against bacterial pathogens and triggered MAPK activation and marker gene expression in the absence of microbes via unbalanced regulatory interactions with its partners (Dominguez-Ferreras *et al.*, 2015). Transgenic lines with high levels of *LecRK-VI.2* expression induce a constitutive PTI response, and microarray analysis of these lines in the absence of microbe infection reveals that the induction patterns of genes in *LecRK-VI.2* overexpression lines are similar to those in SA- or BTH-treated plants (Singh *et al.*, 2012). Priming is the preconditioned state of a plant that promotes stronger and faster responses to stimulation with minimal consumption of energy (Ahmad *et al.*, 2010; Conrath *et al.*, 2015). *CaLecRK-S.5* overexpression *per se* did not trigger MAPK activation, ROS burst, as well as any visible symptoms like HR (Supplementary Fig. S4B–D; 0h). However, transient overexpression of *CaLecRK-S.5* led to induction of defense-related genes in the absence of stress (Supplementary Fig. S5) and promoted stronger MAPK activation and ROS burst in response to wounding treatment compared with empty vector control (Supplementary Fig. S4C, D). These observations also support the conclusion that *CaLecRK-S.5* is involved in priming.

Plant viruses typically initiate infection through wounds caused by mechanical abrasion or by living organisms called vectors such as insects and nematodes, indicating that responses upon wounding stress may be important for plant–virus interaction. The observations that the expression of several genes, categorized into ‘response to wounding’, were diminished in *CaLecRK-S.5*-silenced plants (Supplementary Fig. S6A) and that *CaLecRK-S.5*-overexpressing plants showed stronger activation of MAPK and ROS burst upon wounding stress (Supplementary Fig. S4) suggest that *CaLecRK-S.5* confers sensitivity to wounding stress and that this sensitivity could increase resistance to virus infection. Moreover, the activation of priming by *CaLecRK-S.5* appears to be important for triggering stronger *L*^*2*^ gene-mediated defense responses (Figs 1 and 2).

## Supplementary data

Supplementary data are available at *JXB* online.

The following Supplementary Data is available for this article.

Figure S1. ESTs of *CaLecRKs* upregulated more than twofold during resistance response to TMV-P_0_.

Figure S2. Phylogenetic analysis and amino acid alignment of *C. annuum* LecRK-S.5.

Figure S3. Gene expression pattern of *CaLecRKs* in response to TMV-P_1,2,3_.

Figure S4. Transient *CaLecRK-S.5* expression promotes MAPK activation and ROS burst in response to wounding treatment.

Figure S5. Transient *CaLecRK-S.5* expression induces marker gene expression in *N. benthamiana* leaves.

Figure S6. Transcriptome analysis of *CaLecRK-S.5*-silenced plants compared with empty vector control.

Table S1. List of primers used in this study for RT-PCR, qRT-PCR, and vector construction.

Table S2. Transcriptome pattern in DNA microarray analysis.

Table S3. Functional distribution of ESTs containing kinase domains.

Table S4. Transcriptome pattern in RNA sequencing data.

Table S5. Transcripts upregulated in TRV control by TMV infection but suppressed by *CaLecRK-S.5* silencing.

Table S6. Transcripts downregulated in *CaLecRK-S.5*-silenced plant compared with TRV control plant upon mock treatment.

Supplementary Data

## Abbreviations:

BABA: β-aminobutyric acid
CP: coat protein
DAMP: damage-associated molecular pattern
ETI: effector-triggered immunity
HR: hypersensitive response
ISR: induced systemic resistance
LecRK: lectin receptor kinase
MAPK: mitogen-activated protein kinase
PAMP: pathogen-associated molecular pattern
PMMoV-P_1,2,3_: *Pepper mild mottle virus* pathotype P_1,2,3_
PRR: pattern recognition receptor
PTI: pattern-triggered immunity
ROS: reactive oxygen species
SAR: systemic acquired resistance
TMV-P_0_: *Tobacco mosaic virus* pathotype P_0_
VIGS: virus-induced gene silencing
Xcv: *Xanthomonas campestris* pv. *vesicatoria*.
