# Supplementary material for: CaLecRK-S.5, a pepper L-type lectin receptor kinase gene, confers broad-spectrum resistance by activating priming
Source: J Exp Bot. 2016 Sep 19;67(19):5725–41. doi: 10.1093/jxb/erw336 (PMC5066492; doi:10.1093/jxb/erw336)
Supplement: Supplementary Data [file supp_67_19_5725__index.html]

CaLecRK-S.5, a pepper L-type lectin receptor kinase gene, confers broad-spectrum resistance by activating priming — CaLecRK-S.5, a pepper L-type lectin receptor kinase gene, confers broad-spectrum resistance by activating priming — Supplementary Data 

# *CaLecRK-S.5*, a pepper L-type lectin receptor kinase gene, confers broad-spectrum resistance by activating priming

## Supplementary Data

Data files

- Supplementary\_Figures\_S1\_S6\_Tables\_S1\_S3 - Supplementary Data
- Supplementary\_Table\_S2.xlsx - Supplementary Data
- Supplementary\_Table\_S4.xlsx - Supplementary Data
- Supplementary\_Table\_S5.xlsx - Supplementary Data
- Supplementary\_Table\_S6.xlsx - Supplementary Data
