# Supplementary material for: CaLecRK-S.5, a pepper L-type lectin receptor kinase gene, confers broad-spectrum resistance by activating priming
Source: J Exp Bot. 2016 Sep 19;67(19):5725–41. doi: 10.1093/jxb/erw336 (PMC5066492; doi:10.1093/jxb/erw336)
Supplement: Supplementary Data [file supp_erw336_Supplementary_Figures_S1_S6_Tables_S1_S3.pdf]

The pepper L-type lectin receptor kinase gene *CaLecRK-S.5* confers broad-spectrum resistance by activating priming

Joo Yong Woo, Kwang Ju Jeong, Young Jin Kim, and Kyung-Hee Paek

Woo *et al*. Figure S1

A

Putative function	clone ID	AtDB	fold change
S-locus lectin receptor kinase family protein	PEPPERS0013584	AT4G03230	22.68
lectin receptor kinase family protein	PEPPERS0018100	AT5G60900	16.37
S-locus lectin receptor kinase family protein	PEPPERS0016322	AT2G19130	11.75
S-locus lectin receptor kinase family protein	PEPPERS0002598	AT1G11330	9.49
S-locus lectin receptor kinase family protein	PEPPERS0017189	AT4G27290	7.71
S-locus lectin receptor kinase family protein	PEPPERS0017815	AT4G27300	7.56
lectin receptor kinase family protein	PEPPERS0010681	AT5G06740	4.32
lectin receptor kinase family protein	PEPPERS0018591	AT1G34300	2.18

B

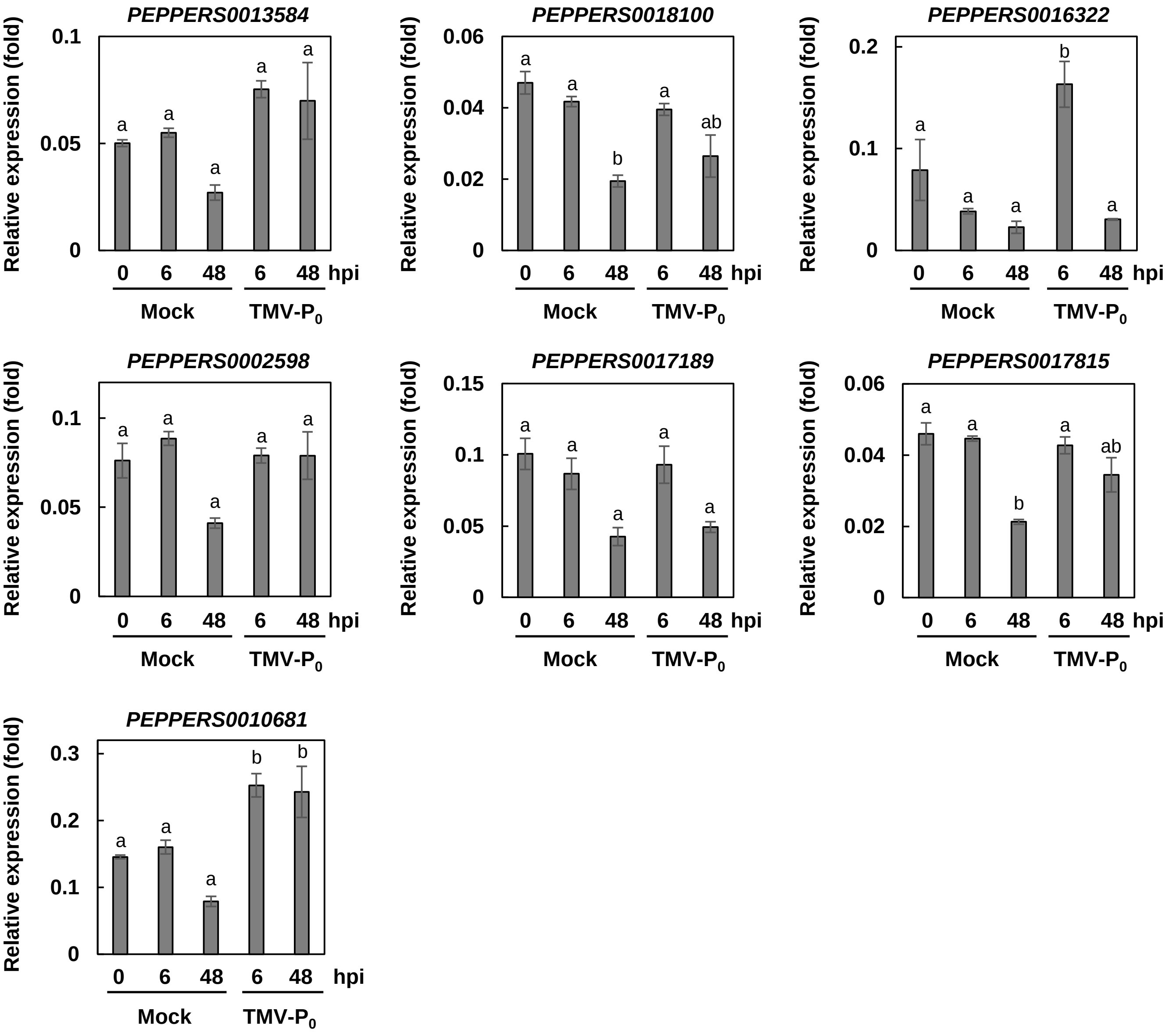

Fig. S1. ESTs of *CaLecRKs* upregulated by more than twofold during resistance response to TMV-P_0. (A) List of selected *CaLecRKs* from microarray analysis. (B) Confirmation of the expression pattern of selected genes by quantitative real-time RT-PCR analysis. Total RNA was extracted from *C. annuum* plants 0, 6, or 48 h after mock or TMV-P_0 treatment. Expression values were normalized to levels of *CaActin* gene expression. Data represent means ± SD of three independent experiments, and different letters indicate significant differences, as determined by one-way ANOVA, followed by Tukey HSD test ($P < 0.01$).

Woo *et al*. Figure S2

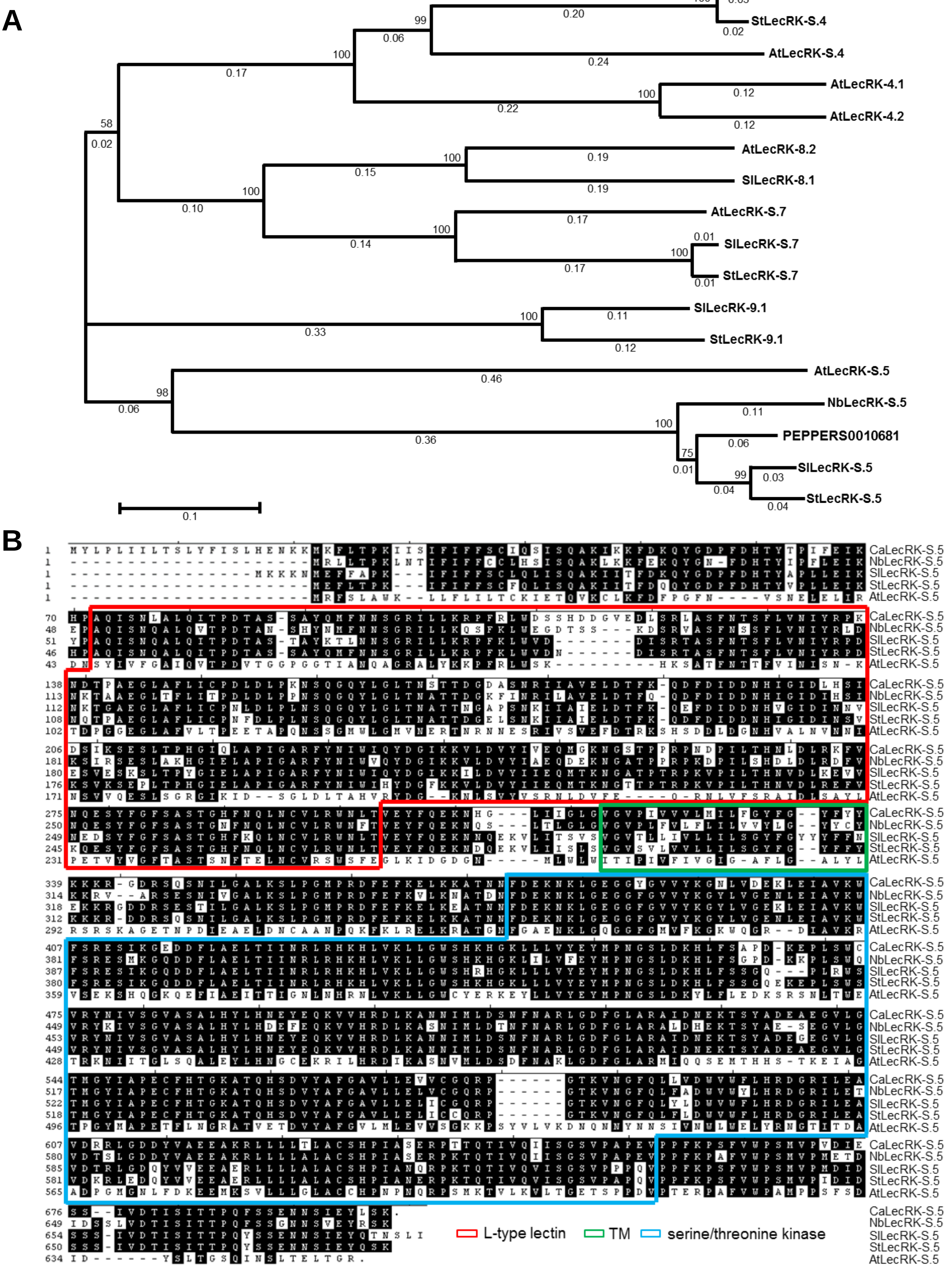

Fig. S2. Phylogenetic analysis and amino acid alignment of *C. annuum* LecRK-S.5. (A) Phylogenetic tree of LecRK amino acid sequences from *Solanum lycopersicum*, SlLecRKs; *S. tuberosum*, StLecRKs; *N. benthamiana*, NbLecRKs; *Arabidopsis thaliana,* AtLecRKs; and *C. annuum*, CaLecRKs. Phylogenetic tree was constructed using the neighbor-joining method (ClustalW) and MEGA4 software. The scale represents amino acid substitutions, and numbers on the tree represent bootstrap scores. (B) Alignment of CaLecRK-S.5 with other LecRK-S.5 proteins from *N. benthamiana* (NbLecRK-S.5, accession no. AKV93699), tomato (SlLecRK-S.5, accession no. XP_004234499), potato (StLecRK-S.5, accession no. XP_006343314), and *Arabidopsis* (AtLecRK-S.5, accession no. NP_196292). Positions of amino acid residues in the corresponding proteins are numbered. Gaps marked with dashes were introduced to maximize sequence alignment. Identical residues were shaded in black. The three domains of LecRK-S.5 were marked with colored boxes.

Woo *et al*. Figure S3

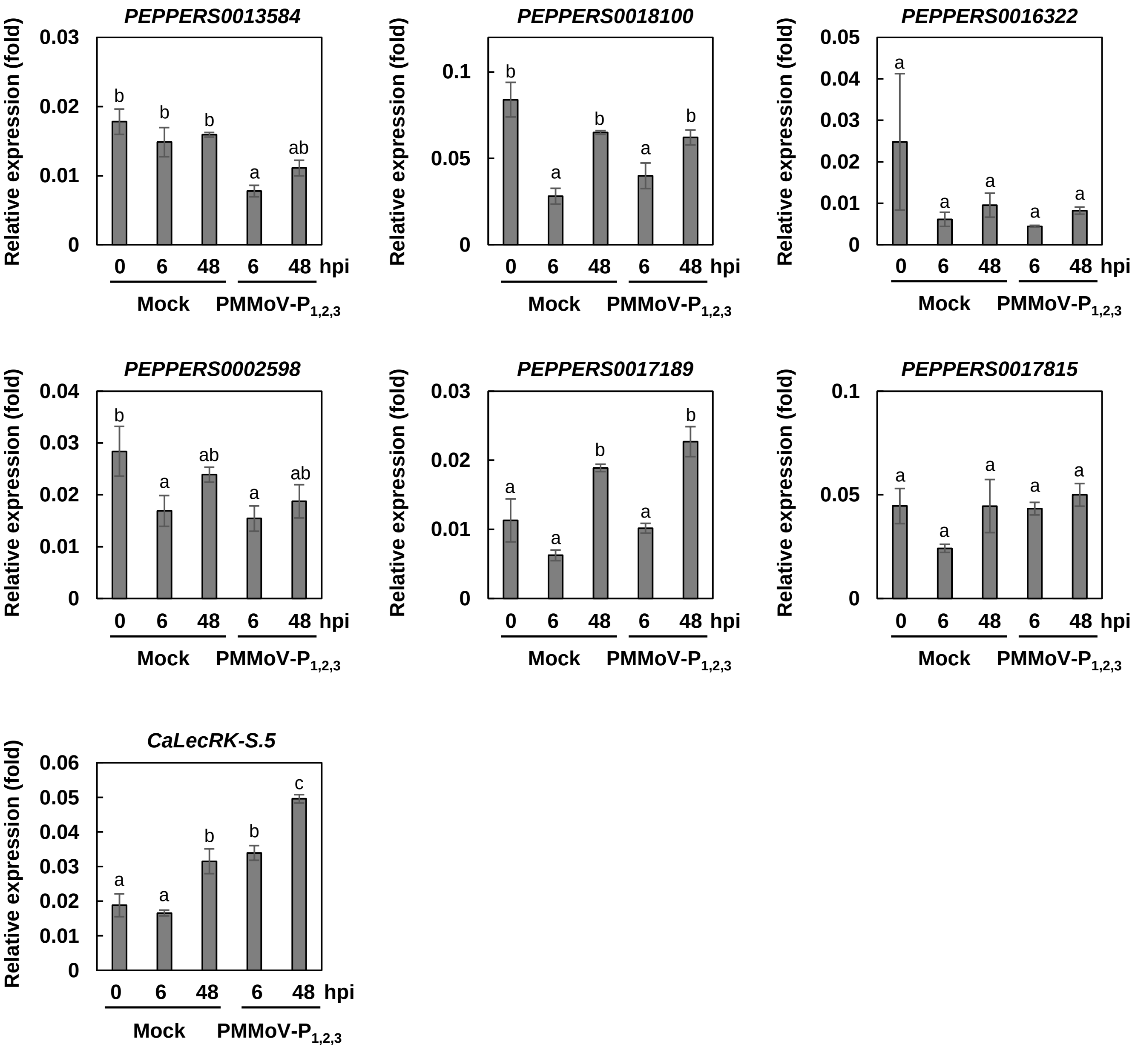

Fig. S3. Gene expression pattern of *CaLecRKs* in response to PMMoV-$P_{1,2,3}$. Total RNA was extracted from *C. annuum* plants 0, 6, or 48 h after mock or PMMoV-$P_{1,2,3}$ treatment. Relative expression levels of *CaLecRKs* were analyzed by quantitative real-time RT-PCR. Expression values were normalized to levels of *CaActin* gene expression. Error bars represent ± SD from three biological replicates, and different letters indicate significant differences, as determined by one-way ANOVA, followed by Tukey HSD test ($P < 0.01$).

Woo *et al*. Figure S4

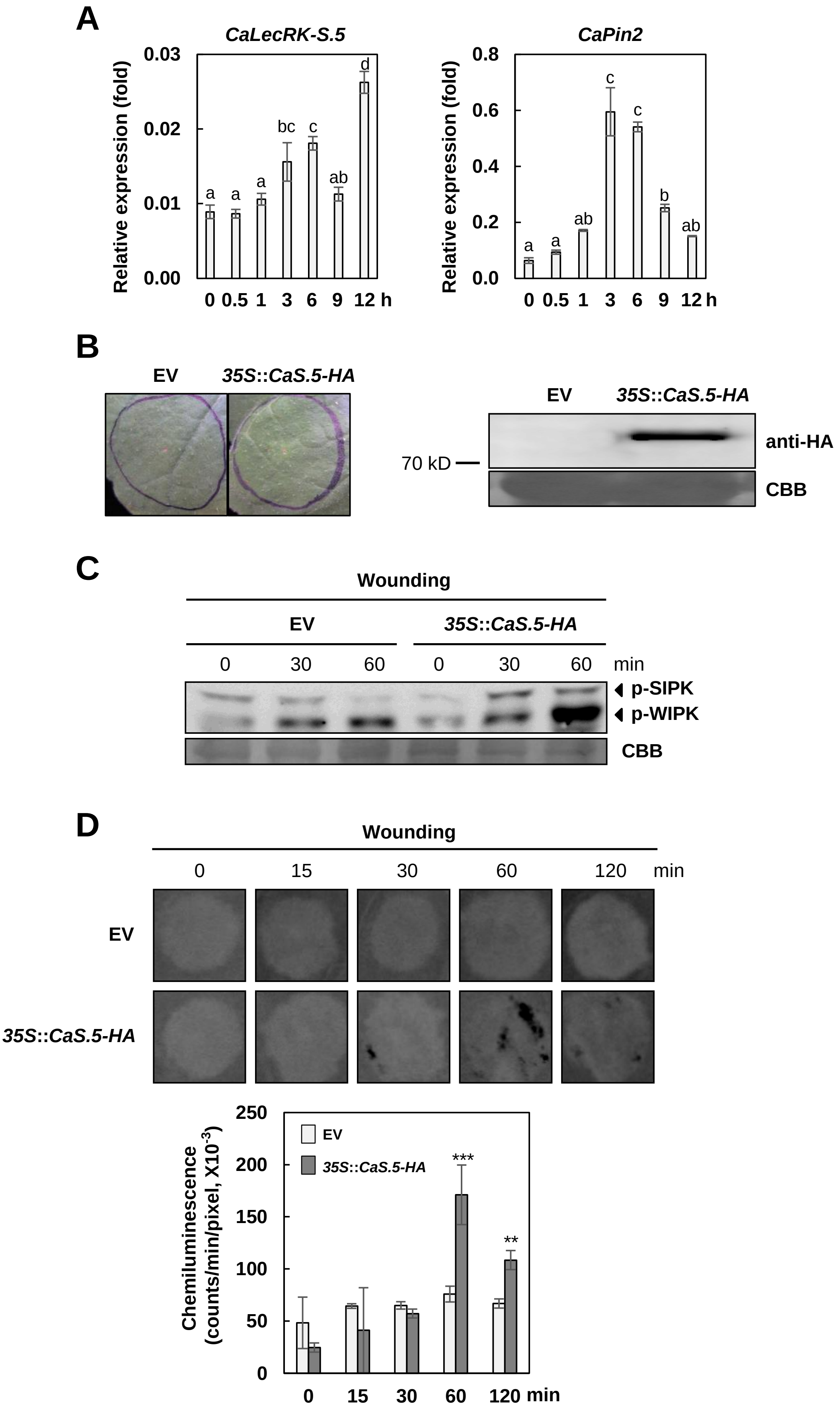

Fig. S4. Transient *CaLecRK-S.5* expression promotes MAPK activation and ROS burst in response to wounding treatment. (A) *C. annuum* plants were treated with wounding stress using carborundum with a cotton swab. Total RNA was extracted from *C. annuum* plants 0, 0.5, 1, 3, 6, 9, and 12 h after wounding treatment. Relative expression levels of *CaPin2*, as a wounding marker gene, and *CaLecRK-S.5* were analyzed by quantitative real-time RT-PCR. Expression values were normalized to levels of *CaActin* gene expression. Error bars represent ± SD from three biological replicates, and different letters indicate significant differences, as determined by one-way ANOVA, followed by Tukey HSD test ($P < 0.01$). (B, C, D) *N. benthamiana* leaves were infiltrated with *A. tumefaciens* carrying empty vector (EV) or CaMV *35S* promoter with *CaLecRK-S.5-HA* (*35S*::*CaS.5-HA*). (B) (left) No difference in phenotype was observed between EV or *35S*::*CaS.5-HA* plants. Photos were taken 3 dpi. (right) Total protein was extracted at 2 dpi. CaLecRK-S.5-HA protein was detected with anti-HA antibody. Coomassie Brilliant Blue staining indicates equal loading. (C, D) Wounding stress was treated 24 h after *A. tumefaciens* infiltration. (C) Phosphorylation of MAPK upon wounding stress in *N. benthamiana* leaves was detected using antibody against phospho-p44/p42 MAPK. Coomassie Brilliant Blue staining indicates equal loading. Wound-induced protein kinase (WIPK) and salicylic acid-induced protein kinase (SIPK), orthologs of *Arabidopsis* MPK3 and MPK6, respectively. (D) ROS production upon wounding stress in *N. benthamiana* leaves was detected by L-012 solution (0.5 mM) and a CCD camera. Chemiluminescence intensities were quantified by Multi Gauge V3.0 (Fujifilm). Data are means ± SD from three experiments (Student's *t*-test, **, $P < 0.05$, ***, $P < 0.01$).

Woo *et al*. Figure S5

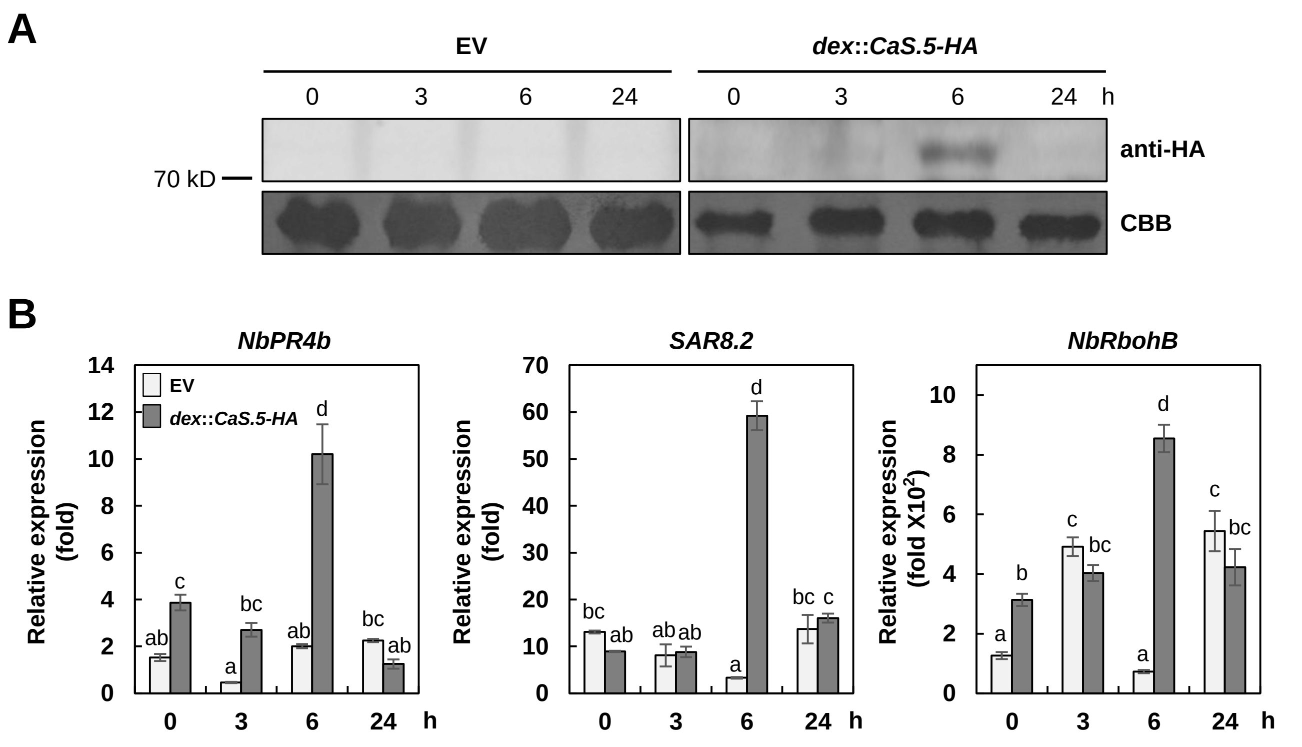

Fig. S5. Transient *CaLecRK-S.5* expression induces marker gene expression in *N. benthamiana* leaves*. N. benthamiana* leaves were infiltrated with *A. tumefaciens* carrying an empty vector (EV) or dexamethasone-inducible *CaLecRK-S.5-HA* (*dex*::*CaS.5-HA*). The leaves were treated with 30 µM dexamethasone (dex) 24 h after *A. tumefaciens* infiltration by a needleless syringe. (A) Immunoblot analysis of CaLecRK-S.5-HA protein using total protein extracts of *N. benthamiana* leaves transiently expressing EV or *dex*::*CaS.5-HA.* Total protein was extracted 0, 3, 6, and 24 h after dex treatment. CaLecRK-S.5-HA protein was detected with anti-HA antibody. (B) Total RNA was extracted 0, 3, 6, and 24 h after dex treatment. Relative expression levels of *NbPR4b*, *SAR8.2*, and *NbRbohB* were analyzed by quantitative real-time RT-PCR. Expression values were normalized to levels of *NbEF-1α* gene expression. Error bars represent ± SD from three biological replicates, and different letters indicate significant differences, as determined by one-way ANOVA, followed by Tukey HSD test ($P < 0.01$).

Woo *et al*. Figure S6

A

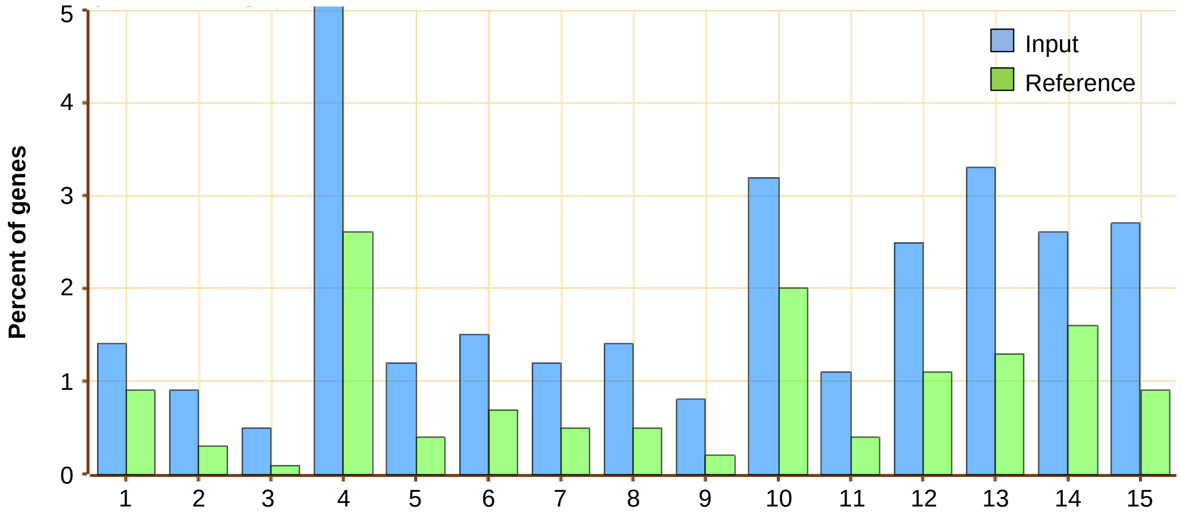

1 : innate immune response
2 : response to extracellular stimulus
3 : response to virus
4 : response to hormone stimulus
5 : defense response, incompatible interaction
6 : response to bacterium
7 : regulation of response to stimulus
8 : response to wounding
9 : response to reactive oxygen species
10 : defense response
11 : response to fungus
12 : response to osmotic stress
13 : response to temperature stimulus
14 : response to light stimulus
15 : response to oxidative stress

B

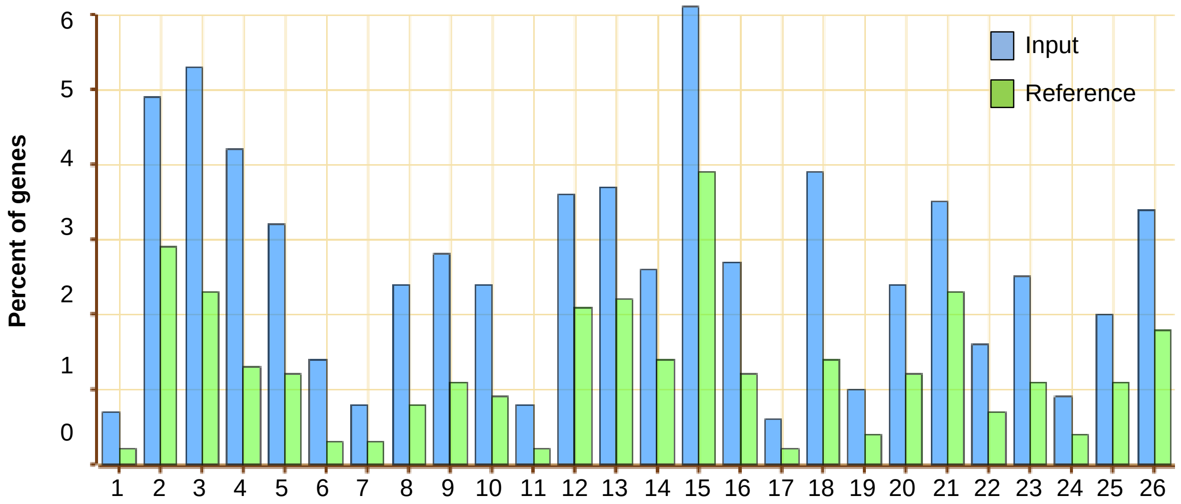

1 : positive regulation metabolic process
2 : phosphorylation
3 : carboxylic acid metabolic process
4 : cellular nitrogen compound metabolic process
5 : cellular response to chemical stimulus
6 : plastid organization
7 : cellular polysaccharide metabolic process
8 : cofactor metabolic process
9 : cellular carbohydrate metabolic process
10 : cellular macromolecule localization
11 : tetrapyrrole metabolic process
12 : proteolysis
13 : lipid metabolic process
14 : amine metabolic process
15 : protein modification process
16 : cellular macromolecule catabolic process
17 : stomatal movement
18 : establishment of localization in cell
19 : pigment metabolic process
20 : heterocycle metabolic process
21 : carbohydrate metabolic process
22 : vesicle-mediated transport
23 : organic acid biosynthetic process
24 : Isoprenoid biosynthetic process
25 : cellular response to stress
26 : cellular amino acid and derivative metabolic process

Fig. S6. Transcriptome analysis of *CaLecRK-S.5*-silenced plants compared with empty vector control. The 2,109 overlapping genes between two groups, genes upregulated by TMV-P_0 infection (compared with mock) in TRV plants and genes downregulated by *CaLecRK-S.5* silencing (compared with TRV) under TMV-P_0 infection, were annotated with IDs in the *Arabidopsis* database (TAIR9) for enriched Gene Ontology (GO) term mapping. (A) Overrepresented GO terms from the 'response to stimulus' category. (B) Overrepresented GO terms from the 'cellular and metabolic process' category. Blue and green colors indicate input and reference, respectively. Y-axis: percentage of genes, X-axis: GO annotation.

Table S1. List of primers used in this study for RT-PCR, qRT-PCR, and vector construction.

Primer	Sequence (5'-3')	Primer	Sequence (5'-3')
PEPPERS0010681 (*CaLecRK-S.5*) F	CCATCACAACACCTCAG TTCA	*PEPPERS0010681* (*CaLecRK-S.5*) R	AAATTCTTTTGACAACCT CACCA
CaLecRK-S.5 VIGS F	TAGCAGCATACATATTA CCAACA	*CaLecRK-S.5* VIGS R	TGCAGCCCAAAAGAAAA
PEPPERS0013584 F	ATCATGCAACAAAGAAGAA GCA	*PEPPERS0013584* R	TGTGTTACCACCACCACAT TTT
PEPPERS0018100 F	CCCAAATCCTTATAGGACT GAAAA	*PEPPERS0018100* R	ACACAAGTGATCAACAA GGCATAC
PEPPERS0016322 F	GTTTGGATAGCAAATAGGG ACAAC	*PEPPERS0016322* R	CATTTGTCAAACAGAGATTT CCAG
PEPPERS0002598 F	TTCACAGGAAGAGATGAAT CTGAA	*PEPPERS0002598* R	CAGAATCCTGCATTTTTC CTATCT
PEPPERS0017189 F	TGCCACGTGATTATTACGA CTC	*PEPPERS0017189* R	AACATCCTTACTTTGGCAG CTC
PEPPERS0017815 F	CTGGAAGATAGACCTAGCA TGTCA	*PEPPERS0017815* R	AAATTGTGATGCTTAATTTG TTCG
CaPin2 F	CGATCCAGATGTAGCTTAC ATGG	*CaPin2* R	GCACAAGGAATGAAACACC
CaNPR1 F	TGAACAGGATTCAATAGAA GTGGA	*CaNPR1* R	AAATCCAGCTCAAGTACCT CATTC
CaWRKY70 F	GGAAGAAGAATCATTAAAG GGACA	*CaWRKY70* R	AAAACTCTATGGCCTCAAA GTCAC
CaWRKY45 F	TCCAAACAAGAAGTCAGGT TGATA	*CaWRKY45* R	AAAATTATCATTGGGCTTGT CAAT
CaPR1 F	GAGGACAACGTCCGTAT GGT	*CaPR1* R	AACTCCAGTTACTGCAC CATTAGA
CaPR2 F	CTACTTAAGCTTTGCAA GACACCA	*CaPR2* R	AGATCTCTTTCCTCATC GTCACTT
CaLTP1 F	GGAAAGAGATCTGTTTTCA TAC	*CaLTP1* R	TCGAAAAGATACACAAAGG AAAG
NbPR4b F	TACAACCCACAGAACATTA ACTGG	*NbPR4b* R	TCTCACTGTTGTTTGAGTTC CTGT
SAR8.2 F	AGTTGATGCAAGGGAGATG TCTA	*SAR8.2* R	AGACTCACACCAGCACA AGTACA
NbRbohB F	TTTCTCTGAGGTTTGCCAG CCACCACCTAA	*NbRbohB* R	GCCTTCATGTTGTTGACAA TGTCTTTAACA
CaLecRK-8.1 F	TGATGTTGATTTGAAAAGT GGTG	*CaLecRK-8.1* R	TGCATCAAATGATGAACTG AAAC
TMV-P_0 CP F	AGCTCGAACTGTCGTACAA AGAC	*TMV-P_0 CP* R	TCTAGTGTCGAATGCACCT AACA
PMMoV-$P_{1,2,3}$ CP F	AAAATCTGTGTACTTCGGC GTTA	*PMMoV-$P_{1,2,3}$ CP* R	GAATCTAGCACGGCATTAT ATCG
CaActin F	GTGCTGAGAGATTCCGT TGC	*CaActin* R	ATGGTTGAGCCACCACT GAG
NbEF-1α F	GAGGGGGACAATATGATTG AAA	*NbEF-1α* R	GTCAGACCAGTAGGTCCAA AGG

Table S3. Functional distribution of ESTs containing kinase domains. Transcriptome of *C. annuum* plants during resistance response to TMV-P_0 infection was analyzed by microarray analysis. Classification of kinases is based on a proposal of Hanks and Hunter (1995).

(Functional distribution) Known or predicted protein	Fold change			TM
	UP (X ≥ 2)	STD (2 > X < -2)	DOWN (X ≤ -2)	
shaggy-related protein kinase	-	6	-	-
cyclin-dependent protein kinase	-	3	1	-
casein kinase	-	4	-	-
ankyrin protein kinase	3	7	-	N
CBL-interacting protein kinase	1	8	3	N
lectin receptor kinase	8	4	1	Y
MAPKKK	2	7	-	N
MAPKK	2	3	-	N
MAPK	4	7	1	N
calcium-dependent protein kinase	9	17	1	N
leucine-rich repeat family protein	20	50	28	Y/N
nonphototropic hypocotyl protein kinase	-	2	-	-
U-box domain-containing protein kinase	-	2	-	-
KCBP-interacting protein kinase	-	1	1	-
glycogen synthase kinase	-	1	-	-
AFC protein kinase	-	2	-	-
SNF1-RELATED PROTEIN KINASE	-	2	-	-
5'-AMP-activated protein kinase	1	5	1	N
3-phosphoinositide-dependent protein kinase	-	2	-	
receptor-like protein kinase	3	3	-	Y
phosphoenolpyruvate carboxylase kinase	-	-	1	
endoribonuclease protein kinase	-	2	-	
AGC kinase	-	-	2	
abscisic acid-activated protein kinase	1	-	-	N
somatic embryogenesis receptor-like kinase (SERK)	2	-	-	Y
peptidoglycan-binding LysM domain-containing protein kinase	-	2	-	
APK2 protein kinase	2	-	-	N
RPM1-INDUCED PROTEIN KINASE	1	-	-	N
cytokinin-regulated kinase	2	-	-	Y
protein kinase family protein, contains protein kinase domain	41	133	17	Y/N
**Total**	102	273	57	
